# *Dictyostelium discoideum:* An Alternative Nonanimal Model for Developmental Toxicity Testing

**DOI:** 10.1093/toxsci/kfab097

**Published:** 2021-08-13

**Authors:** Robert P Baines, Kathryn Wolton, Christopher R L Thompson

**Affiliations:** 1 Department of Genetics, Evolution and Environment, Centre for Life's Origins and Evolution, University College London, London WC1E 6BT, UK; 2 Syngenta, Jealott’s Hill International Research Centre, Bracknell RG42 6EY, UK

**Keywords:** Developmental toxicity, Teratogen, Dictyostelium discoideum, Nonanimal model; social amoeba, genetics, high throughput

## Abstract

A critical aspect of toxicity evaluation is developmental and reproductive toxicity (DART) testing. Traditionally, DART testing has been conducted *in vivo* in mammalian model systems. New legislation aimed at reducing animal use and the prohibitive costs associated with DART testing, together with a need to understand the genetic pathways underlying developmental toxicity means there is a growing demand for alternative model systems for toxicity evaluation. Here we explore the potential of the eukaryotic social amoeba *Dictyostelium discoideum*, which is already widely used as a simple model system for cell and developmental biology, as a potential nonanimal model for DART testing. We developed assays for high-throughput screening of toxicity during *D. discoideum* growth and development. This allowed the toxicity of a broad range of test compounds to be characterized, which revealed that *D. discoideum* can broadly predict mammalian toxicity. In addition, we show that this system can be used to perform functional genomic screens to compare the molecular modes of action of different compounds. For example, genome-wide screens for mutations that affect lithium and valproic acid toxicity allowed common and unique biological targets and molecular processes mediating their toxicity to be identified. These studies illustrate that *D. discoideum* could represent a predictive nonanimal model for DART testing due to its amenability to high-throughput approaches and molecular genetic tractability.

Toxicological safety testing represents a major obstacle for novel pharmaceutical, cosmetic, and agrochemical compounds to reach the market (Brannen *et al.*, 2016). A critical aspect of toxicity testing is developmental and reproductive toxicity (DART) studies, which accounts for more than 10% of preclinical compound failures in the pharmaceutical industry alone (Guengerich and MacDonald, 2007). *In vivo* mammalian testing has been considered the gold standard for DART studies (DeSesso, 2017). However, it is costly, and there is increasing legislative pressure to reduce the number of animals used (DeSesso, 2017; Rovida and Hartung, 2009). Furthermore, there is an increasing appreciation that understanding how each compound exerts its effects at the cellular and molecular level, through the identification of the gene networks affected will enhance future compound safety evaluation. Ultimately, paving the way for toxicity modeling and simulations to become an effective alternative to animal testing (Scialli *et al.*, 2018). Consequently, a variety of alternative whole organism or cell models for DART testing have been developed. They include rodent and human cell assays (Lauschke *et al.*, 2020; Palmer *et al.*, 2013; Panzica-Kelly *et al.*, 2013), rodent tissue assays, and whole embryo culture (Augustine-Rauch *et al.*, 2016). Nonmammalian embryo models include zebrafish and frog assays (Brannen *et al.*, 2016). Each system differs in biological complexity, throughput, predictivity, and the degree to which they can provide links to toxicological mechanisms (Beekhuijzen *et al.*, 2015; Brannen *et al.*, 2016). Consequently it is likely that an integrated strategy using a battery of these assays, together with other newly identified models, will be required to provide information for decision-making that is equivalent to that provided by *in vivo* mammalian development (Brannen *et al.*, 2016).

Many features of the *Dictyostelium discoideum* eukaryotic microbial model system suggest it could play a role in this battery of DART testing models. *Dictyostelium discoideum* is a unicellular eukaryotic amoeba, which feeds via the phagocytosis of bacteria (Williams, 2010). If food is plentiful, it remains unicellular and continues to divide. However, starvation triggers a multicellular developmental process in which thousands of amoebae aggregate together. These cells then undergo cell-type differentiation to form a migratory slug, which contains a small number of cell types that are organized into discrete tissues (Williams, 2010). Finally, after a series of complex and coordinated morphogenetic movements, a fruiting body containing terminally differentiated stalk and spore cells is formed. In a laboratory environment, the full developmental cycle is complete in 24 hours. In practical terms, *D. discoideum* cells are easy to grow to high densities and laboratory strains can be maintained in association with bacteria on agar or tissue culture plates; or in axenic growth medium in tissue culture plates or shaken suspension (Fey *et al.*, 2007). Additionally, growth and development can be largely separated because there is little cell division during development and cells can be induced to develop by simply removing growth medium and plating cells on nonnutrient agar. Thus, reproducible development can be achieved in relatively inexpensive facilities (Fey *et al.*, 2007). Furthermore, *D. discoideum* is amenable to molecular genetic manipulation, including the generation of mutant libraries by restriction enzyme-mediated integration (REMI) (Kuspa, 2006). This has allowed a new method (REMI-Seq) to be developed which allows the relative abundance of each REMI mutant to be determined in complex pools (Gruenheit *et al.*, 2021). Consequently, parallel phenotyping experiments can be performed to identify mutations that affect the responsiveness to selective challenges. Decades of research using these tools in *D. discoideum* have revealed key insights into conserved mechanisms underlying cell motility, chemotaxis, macropinocytosis, phagocytosis, cell-cell signaling, differentiation, and morphogenesis during multicellular development (Dunn *et al.*, 2018; King and Insall, 2009; Loomis, 2014; 2015; Williams *et al.*, 2019). From a DART perspective, this conservation has also allowed *D. discoideum* to be used to identify drug targets and to understand the molecular basis of disease (Cocorocchio *et al.*, 2018; King *et al.*, 2009; Van Driessche *et al.*, 2007; Williams *et al.*, 1999; 2002). *Dictyostelium discoideum* thus represents an attractive, candidate model for DART testing. Despite this, only one study has been performed which sought to evaluate its potential as a model for developmental toxicity screening (Dannat *et al.*, 2003), but the small number of compounds tested made it impossible to draw clear conclusions (Dannat *et al.*, 2003). We therefore set out to develop new high-throughput growth, development, and molecular genetic assays to evaluate *D. discoideum’s* potential as a nonmammalian model for DART studies.

## MATERIALS AND METHODS

###  

####  

##### Study design

This study evaluated the utility of the social amoebae *D. discoideum* to be used in developmental toxicity testing pipelines. Briefly, *D. discoideum* cells were grown or developed in the presence of a total of 37 teratogens and nonteratogens (Supplementary Figure 1 and Supplementary Table 1). A novel high-throughput microscopy assay was established to sensitively measure growth rate and thus define cytotoxic effects (Supplementary Figure 2). Next, developmental toxicity was measured qualitatively and quantitatively. For qualitative assays, samples were blinded and the effects of compounds on developmental morphology or timing were compared by microscopic observation. Quantitative, investigator independent measures of developmental effects were also carried out using a novel assay in which a set of strains containing fluorescent reporters of different developmental stages was used (Supplementary Figure 3). Known cytotoxic and teratogenic compounds lithium and valproic acid (VPA) were used as positive controls, with appropriate solvents used as negative controls to ensure experiment to experiment reproducibility.

##### Selection of test compounds

Test teratogenic and nonteratogenic compounds were selected in a multistep process (Supplementary Figure 1A). To identify a set of test compounds with good evidence supporting their teratogenic effects, as well as diverse physical and chemical properties. We first consulted the catalog of teratogenic agents (Green, 1996) and multiple toxicological databases to identify compounds with U.S. Food and Drug Administration (FDA) pregnancy risk classifications of category C, D, or X or A, B, for teratogenic and nonteratogenic compounds, respectively (defined as “risk cannot be ruled out,” “positive evidence of risk,” and “contradicted in pregnancy,” “controlled studies show no risk” and “no evidence of risk in humans,” respectively). These compounds were grouped based on their teratogenic mechanism. For this each compound was assigned to one of the 6 major teratogenic mechanisms of medical drugs (van Gelder *et al.*, 2010). The 6 mechanisms are “folate antagonism,” “neural crest cell disruption,” “endocrine disruption,” “oxidative stress,” “vascular disruption,” and “specific enzyme/receptor mediated.” Because the first 5 categories provide more specific classifications, we chose several examples from each of these to further refine our list. The final test set of 37 compounds contains 27 teratogenic and 10 nonteratogenic compounds. The final group of test compounds exhibit diverse physical and chemical properties (Supplementary Figure 1B). Furthermore, a selection of the compounds have been used in previous studies to address the utility of other model systems, thus allowing us to cross compare results obtained in *D. discoideum* (Ali *et al.*, 2011; Ducharme *et al.*, 2015; Panzica-Kelly *et al.*, 2013).The test compounds represent a range of different physical chemical properties with molecular weight range of 42.39 and 598.08 g/mol, which corresponds to a range in calculated structural complexity (2–839). The polar surface area of the compounds ranges between 0 and 230. The compounds are equally split between H_2_O and dimethyl sulfoxide (DMSO) as primary solvents with 17/37 (45%) and 18/37 (48%), respectively. All compounds were procured from Sigma-Aldrich.

##### High-throughput time lapse microscopy-based growth assay

A simple high-throughput microscopy method was devised to measure growth rate. *D. discoideum* cells rapidly attach to the substratum in tissue culture plates (Supplementary Figure 2A). This allows the number of cells over time to be monitored by time lapse microscopy and when a programmable microscope stage and multiwell plates are used, the effects of multiple compounds or concentrations can be assayed simultaneously (Supplemenmtary Figure SA). *D. discoideum* Ax4 cells were maintained in HL5 growth media (1% peptone, 0.72% yeast extract, 1.54% glucose) supplemented with Penicillin G, Streptomycin sulpfte, folate, and vitamin B12 or maintained on a lawn of *Klebsiella aerogenes* (*Ka*) on standard media plates (1% glucose, 1% peptone, 0.1% yeast extract, 2% agar) (Fey *et al.*, 2007). For the assay, Ax4 cells were collected from HL5 medium during exponential growth and resuspended in fresh HL5 at a concentration of 1 × 10^4^ cells per ml with either 100 μl or 1 ml plated per well in 96 or 24 well plates, respectively. Cells were allowed to settle for 20 min before filming at 4× magnification using an Olympus IX71 microscope augmented with a programmable automated stage (Prior). 96 well plates assays were conducted using 3 replicate wells for each sample. Methods for the automated recognition and counting of cells were optimized (Supplemenmtary Figure 2B). Images from individual growth films were stacked and inverted using ImageJ software (Fuji). Cell number per frame over the whole of the inverted film was extracted using the “trackmate” plug-in for ImageJ (Tinevez *et al.*, 2017). The number of cells per frame was plotted to generate a growth curve from which the doubling time during the exponential growth phase was calculated (generally between 8 and 48 h). No significant difference was seen in growth rates between different positions within a well, or between wells (Supplementary Figure 2C). The minimum number of images that were required without compromising the accuracy of growth rate was found to be at a 1-h framerate (Supplementary Figure 2D). To test the effects compounds had on growth rate, 3 replicate wells were set up for each sample and a maximum solvent concentration of 1% was used to ensure that control samples did not exhibit growth defects. The initial dose range for characterizing the effects of the test compounds on cell growth was based on an “anchoring” starting dose which was selected by converting the rat LD_50_ mg/kg value for each compound to a molar concentration. The rat LD_50_ was selected from either intravenous or intraperitoneal administered treatment as the direct absorption of chemicals into the bloodstream is intuitively most similar to adding compounds directly to *D. discoideum* growth media (Cassar *et al.*, 2019). Once this dose was identified, a dose range above and below the anchor dose concentration was tested. This initial testing dose range spanned from 4- to 1000-fold depending on the variability of the published initial anchor dose (data not shown) and the solubility of each compound. Once the growth no-observed-adverse-effect level (NOAEL) or lowest-observed-adverse-effect level (LOAEL) threshold had been identified (usually in the first pass) up to 2 more experimental dose ranges were tested to narrow down the NOAEL/LOAEL values.

##### Qualitative *D. discoideum* developmental toxicity assessment

Trained researchers typically use key developmental milestones to score the effects of a compounds’ exposure on timing or morphology. This begins with the aggregation of cells that form tipped mounds, which in turn form a slug, a culminant, and finally a terminally differentiated fruiting body containing stalk and spore cell types. A dose range was defined for each test compound using the NOAEL growth toxicity dose as a middle anchor dose. From this dose, 3 doses (5-, 25-, and 125-fold) lower and two (5- and 25-fold) higher doses were assessed. For development, exponentially growing cells from axenic media were harvested during log phase of growth and washed twice in KK2 buffer (16.1 mM KH_2_PO_4_, 3.7 mM K_2_HPO_4_). Cells were resuspended in KK2 at a concentration of 4 × 10^7^/ml before 5 or 20 μl of cell suspension was spread evenly across the surface of a well of a 96- or 24-well dish containing 100 μl or 1 ml of 1.5% KK2 nonnutrient agar as the developmental substratum. All plates were incubated in the dark at 22°. The developmental toxicity assessments were conducted with duplicate wells for each dose assayed. The compounds were dissolved into the agar prior to the experiment with a maximum solvent concentration of 0.5%. The developments were visually inspected after 4, 8, 12, 16, 20, 24, and 48 h (timings of the major stages of development). The experimental developments were repeated up to 3 times over different weeks with only consistent toxic phenotypes recorded. Representative images were recorded at the mound, slug, and/or fruiting body stage using a Lecia MZ-16-FA dissecting microscope with a Hamamatsu camera and HCImageLive software.

##### High-throughput fluorescence plate reader-based developmental toxicity assay

Manual scoring of developmental effects is time consuming, subjective, and restricted to expert laboratories. A high-throughput assay that provides a quantitative and reproducible read out of the key transitions and cell fate decisions throughout the *D. discoideum* developmental cycle was therefore developed. Developmental stage fluorescent reporter strains were constructed by integrating the gene promotor regions of developmental expressed genes into the extrachromosomal RFP expression plasmid, pDM324 (Veltman *et al.*, 2009). To identify reporter genes for the major stages of *D. discoideum* development, published RNA sequencing data from a developmental time course was used to identify genes that are expressed at specific stages (Rosengarten *et al.*, 2015). Only genes with a comparatively high expression and an expression peak of in which >30% of total developmental expression was restricted to a single time point were selected. Six genes were chosen that represent makers of different stages of development (Supplementary Figure 3Ai). These genes (DDB_G0273495, DDB_G0273641, DDB_G0286321, DDB_G0280847, DDB_G0270722, and DDB_G0274335) were selected as developmental imitation, streaming, mound, slug, culminant, and fruiting body reporters. The promoters of these genes were amplified, cloned into RFP expression vectors and transformed into *D. discoideum* cells (Veltman *et al.*, 2009). In addition, cells lines were generated that express GFP or RFP under the control of known promoters of the prespore and prestalk cell-type markers, pspA and ecmA (Supplementary Figure 3Aii). Transformed strains were generated by electroporation and selection in G418 (20 μg/ml), which was removed 48 h prior to carrying out developmental assays (Veltman *et al.*, 2009). All experimental developments were set up in 96-well plate format using black opaque 96-well “visionplates” (4titude), which were found to reduce the background fluorescent signal in all conditions tested. Three individual developments were conducted for each reporter strain at every dose tested. Test compounds were dissolved in the agar prior to developmental exposure, with a maximum solvent concentration of 0.5%. A Synergy H1 (Biotech) multimode plate reader was used to measure fluorescent signal as adjustable *Z* focus and the ability for a top-down read were technical features found essential to detecting a signal from developments. GFP and RFP signals were measured using 485/528, 532/610 excitation/emission wavelengths, respectively. Developments conducted within the plate reader were incubated at 22°C with readings taken every 2 h. After every fluorescence plate reader assay, plates were manually inspected to assess whether the control developments had progressed normally. Fluorescent reporter data for each time point was normalized to the total fluorescent signal over the 24 h time course for each replicate. The data were considered to have a developmental profile if the mean value of any of the time points had a value 1.1 to 1.6-fold (dependent on each stage reporter) greater than the mean of the total fluorescent signal over the 24 h time course. The fold change was selected as the highest threshold in which a peak in signal was always observed in every control development. By comparing the timing and fluorescent value of the peaks between the control and compound treated developments, using the 1.1 to 1.6-fold threshold, differences in developmental timing and/or heterogeneity was statistically assessed. Statistical differences in the timing and strength of the fluorescent values were assessed using t tests. These data reveal the “development initiation” reporter is maximally expressed at the beginning of development, after which expression decreases as development proceeds (Supplementary Figure 3Bi). The “streaming” and “mound” reporter fluorescence can be detected from approximately 6 h and 12 h respectively, and the signal continues to rise throughout development (Supplementary Figure 3Bii, iii). A strong fluorescent signal is observed from the slug reporter throughout development, but the signal transiently rises sharply between approximately 12–16 h (Supplementary Figure 3Biiii). Both the “culminant” and “fruiting body” reporters are only expressed toward the end of development (Supplementary Figure 3B). The culminant reporter begins to be expressed after approximately 20 h and the fruiting body reporter is only expressed after 22–24 h. Together, with cell-type reporters (Supplementary Figure 3Bvii, viii), the stage reporters can be used to measure progression through key developmental stages from vegetative growth to cell-type specification and fruiting body formation.

##### Collection of *in vivo* rat and alternative model toxicity datasets

Four *in vivo* rat toxicity datasets were collected for this study: acute toxicity (LD_50_), repeat dose toxicity (subacute), repeat dose toxicity (subchronic), and developmental toxicity (rat fetal teratogenicity). Only data from the oral administrative route were collected and 100% bioavailability was assumed for all compounds. All values were converted to molarity from mg/kg doses assuming that 1 kg of mammalian body weight is equivalent to 1 l (Ducharme *et al.*, 2015). Acute toxicity data were collected using LD_50_ values. Values were obtained using the FDA drug registration label. Values were also collected from both ChemIDplus and the Hazardous Substances Data Bank (HSDB). If there were any inconsistencies between values obtained from difference sources the lowest value was used. Rat repeat dose toxicity values were split into 2 datasets dependent on the dosing length of the study: “subacute” for studies ≥7–≤28 days and “subchronic” for studies 3–6 months. An extensive literature search was conducted to collect the repeat dose toxicity values: with toxicity databases (ChemIDplus, Pubchem, HSDB), research papers, FDA drug registration labels, and manufacture’s material safety data sheets and product monographs used in collecting the values. Where possible the NOAEL and LOAEL values defined in each studies observation were recorded. For the rat developmental toxicity dataset, the LOAEL value for teratogenicity in rat fetus was collected. Only LOAEL doses were collected for the developmental toxicity endpoint as not all studies achieve a NOAEL. Doses associated with the induction of reversible or minor manifestations of developmental toxicity (eg, changes in fetal weight, growth suppression) were not used for this assessment. Values were obtained using the FDA drug registration label with some of the nonteratogenic control compound values obtained from UK committee toxicity report. A Zebrafish embryo acute toxicity (LC_50_) dataset was collected from Ali *et al.* (2011). A larger composite zebrafish embryo developmental toxicity dataset was created by combining LC_50_ and LOAEL values collected from Ali *et al.* (2014) and Ducharme *et al.* (2015), respectively. Mouse embryonic stem cell viability (IC_50_) values were collected from Panzica-Kelly *et al.* (2013). Where there was a sufficient sample size (>3) Pearson correlation analysis was performed to identify the significance, *R* and *R*^2^ values between every toxicity endpoint (Graphpad—Prism).

##### *Dictyostelium discoideum* teratogen predictivity performance analysis

A teratogenic potential ratio analysis was used to classify compounds in *D. discoideum* as teratogenic or nonteratogenic (Ball *et al.*, 2014; Panzica-Kelly *et al.*, 2010). This is based on the idea that differences in the ratio between doses that result in general toxicity (LD_25_ in zebrafish) versus developmental toxicity (Developmental toxicity NOAEL) can be used to classify compounds. Performance is quantitated by assessing the percentage of “true positive” or “false negative” and “true negative” or “false positive” in relation to the mammalian classification for teratogens and nonteratogens, respectively. From these classifications endpoints for the sensitivity for detecting teratogens, specificity for detecting nonteratogens, positive predictive value, negative predictive value, overall predictivity, and overall concordance can be calculated (Ball *et al.*, 2014). To assign similar values in *D. discoideum*, for each compound we used the growth toxicity LOAEL as a proxy for the LD25, together with the *D. discoideum* developmental toxicity NOAEL. Where a growth toxicity LOAEL could not be defined for a subset of the test compounds the NOAEL values were used in its place. The ratio analysis was performed for all 37 test compounds and excluding the 13 test compounds that could not be assigned a growth toxicity LOAEL value (Table 1). A growth LOAEL/development NOAEL ratio of ≥10 was considered a classification for a teratogen and ≤10 for a nonteratogenic classification. This ratio provides the best threshold for *D. discoideum* performance. A similar approach was taken in Zebrafish where a ratio of ≥10 was also used to classify compounds as teratogenic (Ball *et al.*, 2014; Panzica-Kelly *et al.*, 2010).

**Table 1.** Summary of the Performance of *D. discoideum* to Predict Teratogenicity

Assay Result	Teratogens	Nonteratogens	Sensitivity^*a*^	Specificity^*a*^	Positive Predictive Value^*a*^	Negative Predictive Value^*a*^	Overall predictive value^*a*^	Overall concordance^*a*^
All compounds	27	10	67	80	90	47	69	70
Excluding compounds without a growth LOAEL	19	5	79	60	83	43	66	75

a Values are percentages.

##### REMI-Seq selection

REMI-Seq pools containing approximately 23 000 mutants were hatched and split in two 10 cm tissue culture plates in standard HL5 media (Gruenheit *et al.*, 2021). The pool was briefly allowed to recover and proliferate for 24 h with care taken not to bottleneck the population. The pool was allowed to grow to confluency (approximately 3 × 10^6^/ml) before being split into 3 populations for setup of the 7.5 mM lithium, 1 mM VPA, and 1% DMSO screens. Each screen was conducted with 2 biological replicate populations. For each biological replicate (for each screen), three 10 cm tissue culture plates were seeded at 2 × 10^5^/ml and grown until confluency (approximately 3.5 generations). At which point the 3 plates of each biological replicate were pooled, counted, and reseeded into 3 new plates at 2 × 10^5^/ml. The remaining cells were frozen down into multiple aliquots. This process constituted a single round for the screens and was repeated 5 times.

##### REMI-Seq gDNA sample processing and sequencing

Genomic DNA was obtained from cells from both replicates of rounds 2 and 5 for the DMSO, lithium and VPA screens. Frozen pool samples (25 μl suspension of 2.5 × 10^5^ cells) were thawed directly into 400 μl of an overnight culture of *Ka* and plated on a SM plate. The cells were grown overnight at 22°C until a clearing plate had formed but before *D. discoideum* development structures had formed. Nuclei were collected from approximately 5 × 10^8^ cells per sample that had been washed 6 times in 4°C KK2 (removing residual *Ka* cells) and resuspended in 30 ml nuclei buffer (40 mM Tris, pH 7.8, 1.5% sucrose, 0.1 mM EDTA, 6 mM MgCl_2_, 40 mM KCl, 0.4% NP-40 substitute, 5 mM DTT). The suspension was centrifuged at 4000 g for 30 min, 4°C. The supernatant was discarded leaving pellets. The pellets were suspended in EDTA to a final concentration of 100 mM before adding 10% sodium lauryl sarcosyl (SLS) mixing and incubating at 55°C for 20 min. 4 M ammonium acetate (250 μl) was added and the mixture was centrifuged at 20 000 g for 15 min at 4°C. One volume of supernatant was added to 2 volumes of 100% ethanol, mixed and centrifuged at 20 000 g for 10 min, 4°C, from which the supernatant was discarded. The pellets were washed with 70% ethanol, dried and suspended in 50 μl of 10 mM Tris pH 7.8, containing RNase A and RNase T1 (10 U/ml and 400 U/ml respectively, Ambion). Finally, the gDNA samples were visualized by electrophoresis on a 1% agarose gel. Each gDNA sample was prepared specifically for REMI-Seq sequencing as described by Gruenheit *et al.* (2021). Briefly, the samples were digested with Mmel and I-SceI excising a DNA fragment contain the junction of the gDNA and REMI insert. Indexed adapters (D7 and D5) were ligated to the digested DNA. Different combinations of D7 or D5 indices were used to tag the individual samples for each screen and biological replicate. The DNA fragments were amplified by PCR, using primers specific to the ligated adapters. The samples were separated by gel electrophoresis and the resulting DNA fragments were excised and quantified using a Qubit 3.0 Fluorimeter (ThermoFisher). Sample from rounds 2 and 5 were sequenced separately using a Illumina NextSeq 500 Sequencer with a High Output Kit v2 (75 cycles).

##### Sequencing data processing and identification of enriched or depleted mutants

The sequencing data were processed for REMI-Seq analysis as described by Gruenheit *et al.* (2021). The raw read counts were normalized using the total number of reads per sample and the total number of reads per insertion point. Insertion points and tags that could not be uniquely assigned to one position were removed. The analysis was performed on the round 2 and round 5 samples, separately. Following the sequencing data analysis, mutants were binned according to their mean normalized DMSO replicate read counts for the round 2 and round 5 samples (bin 100 = <100 reads, bin 1000 = 100−1000 reads, bin 10 000 => 1000 reads). Next, the log fold-change values relative to DMSO replicate mean read count were calculated for each insertion mutant. This was completed for both replicates of the lithium and VPA screen, for rounds 2 and 5. Normalized REMI-Seq data are available at https://doi.org/10.5061/dryad.z612jm6cb (last accessed August 16, 2021). Mutants with a *Z*-score > 1.5 in each biological replicate for the lithium or VPA screens were considered to have an advantage. Similarly, mutants with a *Z*-score < −1 biological replicate for the lithium or VPA screens were considered to have a disadvantage. Mutants with fewer than 100 read counts in the DMSO screen were discounted from the disadvantage analysis because the technical dropout rate for these mutants is very high (Gruenheit *et al.*, 2021). Mutants with intergenic insertions that were <500 bp upstream of a gene were assigned to that corresponding gene. Mutants were removed from the significantly advantaged or disadvantaged lists if they were found to have gene inserts in tRNAs, pseudogenes, or transposable genetic elements.

##### Growth competition assay

Pools of REMI-Seq mutants or individual REMI-Seq mutants from the REMI-Seq-Bank (Gruenheit *et al.*, 2021) were assessed in a growth competition assay. REMI-Seq mutants with insertions in lithium or VPA screen identified genes were hatched from the REMI-Seq mutant bank (Gruenheit *et al.*, 2021). A DpoA single gene deletion strain and its parental Ax2 cell line were obtained from the Dictybase stock center. Ax4-GFP cells and the competitor cells were harvested from tissue culture conditions and mixed together 50:50 to a cell density of 2 × 10^5^/ml and seeded into duplicate wells of a 24-well plate. The mixed cell populations were assayed with the addition of 1% DMSO and 7.5 mM lithium or 1 mM VPA with 2 technical replicates per condition. Cells were allowed to grow together until confluency (approximately 3.5 generations), mimicking the conditions of each of the screen rounds. The relative proportion of GFP labeled to unlabeled cells was scored at the start as well as the end each round of the competition assays by flow cytometry (Attune NxT Flow cytometer). The competitions were continued until either the labeled or unlabeled cells were at 100% or 6 rounds were completed. The competition data were normalized to the expected starting frequency (50:50). The competitions were further normalized as a ratio of the test mutant to GFP-labeled cells (0–1). For validation of the putative advantage and disadvantage mutants, the mean log fold change between the normalized drug and the nondrug-treated competitions at rounds 3 and 6 (or the final round tested) was used to generate a competition fitness score.

##### Gene ontology term analysis

Gene ontology **(**GO) term analysis was performed using the GSEAbase R package (Morgan *et al.*, 2021). A cutoff of *p* = .05 was used for significantly overrepresented biological process GO terms. Gene lists containing 173 lithium genes (round 2 disadvantaged and round 5 advantaged), 235 VPA genes (round 2 disadvantaged and round 5 advantaged) and both lists combined (376 genes) were compared against a universe of genes from every mutant in the starting library and all detectable mutants in either round 2 or 5 of the screen. The universal gene list was also modified to remove all gene excluded from the reference lists (tRNAs, pseudogenes, transposable genetic elements, and nonpromoter intergenic insertions). After the GSEAbase analysis, the significant GO terms for biological process were simplified using the REVIGO tool (Supek *et al.*, 2011). The redundancy of the GO terms was calculated and >0.5 threshold used to remove the most redundant terms (Supek *et al.*, 2011). The overrepresented genes underlying the significant biological process GO terms were classified as either coming from the lithium and/or VPA screens. Then the biological process GO terms were classified as either uniquely lithium, VPA or common, dependent on the origin of the genes underlying them. The biological process GO terms were also classified into 4 major categories: “metabolic process,” “signal transduction,” “response to stimulus/stress/DNA damage,” and “vesicular transport,” dependent on where they clustered on the GO term tree.

##### Fluid uptake assay

A fluid uptake assay was performed as described by Williams and Kay (2018), but modified for a 24-well plate format. REMI-Seq mutants from the REMI-Seq-Bank and a control REMI-Seq mutant with a neutral intergenic insertion were tested using the assay. For each condition assayed, 1 × 10^5^ axenically growing cells were plated in triplicate in the wells of a 24-well plate. After settling in the wells for 20 min, 7.5 mM lithium or 1 mM VPA was added to noncontrol cells and the plate was incubated at 22°C for 23 h. After 23 h the HL5 media was aspirated and the cells incubated for 1 h with 0.5 mg/ml TRITC-dextran (Sigma-Aldrich). A total of 7.5 mM lithium or 1 mM VPA was added to noncontrol cells during the incubation period. After 1 h the TRITC-dextran was aspirated, the cells quickly washed in cold KK2. After which the cells from each well were collected in 1 ml ice-cold KK2 + 5 mM sodium azide (preventing exocytosis). Median fluorescence intensity was measured by flow cytometry (Attune NxT Flow Cytometer). All values were normalized to each mutant’s control, with at least 2 independent experiments performed per mutant. A fluid uptake score was assessed by first calculating the foldchange reduction in fluid uptake after treatment of lithium or VPA in both the control mutant and test mutant. The log fold change between the mutants and control mutants’ fold change reduction was calculated as a fluid uptake score.

## RESULTS

###  

#### High-Throughput *D. discoideum* Growth and Developmental Toxicity Assays

**Figure 1.**
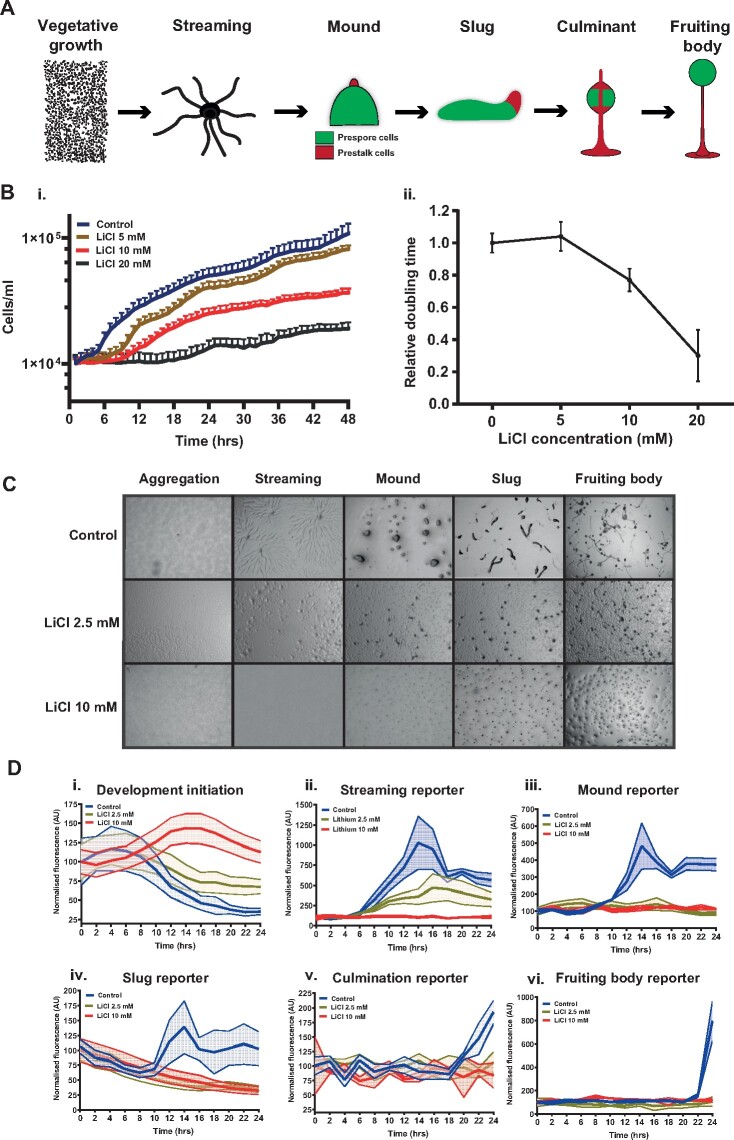
New high-throughput methods to measure growth and developmental toxicity in *Dictyostelium discoideum*. A, Schematic of the *D. discoideum* developmental cycle. Vegetative amoebae undergo a developmental cycle upon starvation. Streams of cells aggregate to form a spherical mound, where cells differentiate into either prestalk or prespore cell types. The prestalk cells form a tip on the mound which extends, forming a multicellular migratory slug. Eventually the slug halts forming a culminant structure, finally resulting in a fruiting body. The fruiting body is composed of terminally differentiated stalk and spore cells. B, Chemical toxicity can be accessed via relative change in cell doubling rates. Growth curves (i) and the relative doubling time (ii) of Ax4 cells when treated with 5, 10, and 20 mM LiCl (mean ± SD). C, Lithium treatment halts *D. discoideum* development before the streaming stage. Ax4 cells were developed for 24 h in the presence of either 0, 2.5, or 10 mM LiCl. Images are representative of multiple independent experiments. D, Lithium-induced developmental toxicity can be assayed using fluorescent reporters. Fluorescent reporter readouts for the major stages of development: development initiation, streaming, mound, slug, culminant, and fruiting body over 24 h in the presence of 2.5 mM or 10 mM LiCl (mean ± SEM).

To evaluate the potential of *D. discoideum* as a predictive model for DART studies 37 test compounds (27 known teratogens, 10 nonteratogens were chosen) (see Materials and Methods and Supplementary Figure 1 and Supplementary Table 1). To measure dose-dependent effects on growth, we developed a new high-throughput assay in which growth rates are measured by time-lapse microscopy (see Materials and Methods and Supplementary Figure 2). Effects on development were first measured by qualitative monitoring of developmental progression (timing and morphology) through key morphological transitions (Figure 1A). Furthermore, to quantitatively measure effects on development we also developed a new high-throughput assay based on stage-specific fluorescent reporter strains, which does not require *D. discoideum* expertise and is independent of observer bias (see Materials and Methods and Supplementary Figure 3). The growth and quantitative development assays were validated by testing the effects of lithium chloride (LiCl) (a known cytotoxic and teratogenic agent) (Pastor *et al.*, 2009; Patorno *et al.*, 2017; Williams *et al.*, 1999). During growth, LiCl toxicity has been reported to occur at dosages >6 mM in mammalian cells, with concentrations above 10 mM reported to cause cytotoxicity in both *D. discoideum* and different mammalian cell types (King *et al.*, 2009; Pastor *et al.*, 2009; Repetto *et al.*, 2001; Williams *et al.*, 1999). Growth rates in LiCl were compared with untreated control cells over 48 h (Figure 1Bi). Concentrations of LiCl up to 5 mM did not affect growth rate. However, concentrations of 10- and 20-mM reduced growth rates significantly (Figure 1Bii). At these doses (>10 mM), LiCl also severely affects development, resulting in a failure to form slugs or fruiting bodies within 24 h (Figure 1C). As expected, at the 10 mM LiCl dose, expression of the development initiation reporter remained high and did not fall over the whole-time course, whilst later markers were not induced (Figure 1Di). At lower doses (approximately 2.5 mM), aggregation is visibly delayed with partial, localized streaming of cells, again resulting in a failure to form fruiting bodies within 24 h (Figure 1C). As expected, in 2.5 mM LiCl, the development initiation reporter shows a similar temporal pattern to the control (although expression is slightly higher at all time points) and the streaming reporter does activate (but the signal is less than the untreated control [Figure 1Dii]). However, the late developmental reporters are never activated (Figure 1C). These results thus demonstrate that the growth and quantitative development assays provide simple high-throughput quantitative methods to assess toxicity during growth and development, which require little prior expertise in *D. discoideum* biology.

#### Measurement of Growth and Developmental Toxicity in *D. discoideum*

Because most of the 37 selected compounds have never been tested in *D. discoideum*, we first determined their effects on growth, as this provides the simplest readout for toxicity. Growth rates were determined using the microscope-based growth assay over a 48-h period. We defined dose ranges that spanned (where possible) the NOAEL/LOAEL toxicity threshold during the first experimental pass. Subsequent experiments were then performed to refine these values by narrowing the dose range around the NOAEL/LOAEL toxicity threshold. We were able to define both a NOAEL and LOAEL value for 24 of the 37 test compounds. However, the maximum solubility for 13 compounds meant that only a NOAEL value could be defined. For 8 of these compounds (primidone, cyclophosphamide, cisplatin, cefotaxime, ascorbic acid, acebutolol, penicillin G, and metformin) growth toxicity was not seen at the highest soluble dose. The remaining 5 compounds (phenytoin, 13-cis-retinoic acid, retinoic acid, bosentan, and bexarotene) precipitated in the growth medium, which may limit exposure. Consequently, a NOAEL was recorded for all 13 of these compounds with a solubility caveat, because they are likely an underestimation or overestimation, respectively.

Having defined NOAEL and LOAEL (where possible) values for each compound during *D. discoideum* growth, we next investigated their effects during development. All compounds were tested at the growth NOAEL dose, as well as 3 lower doses (5, 25, and 125-fold). 22 compounds could also be tested at a 5-fold higher concentration, whilst 12 of these could also be tested at a 25-fold higher concentration. Whilst this first pass could easily be performed using the high-throughput plate reader assay, to expedite this process, we performed a visual “qualitative” assessment of developmental toxicity. The fluorescence plate reader assay was then used to further refine developmental LOAEL and/or NOAEL values. This dual approach also enabled us to use the quantitative fluorescence plate reader assay to validate the subjective nature of our manual qualitative assessments; as well as providing further validation of the utility of the fluorescence plate reader assay.

For the qualitative dataset, the timing and morphology of the major stages of development was noted over 24 h with a final observation also taken at 48 h in order to allow severe developmental delay to be scored. The qualitative developmental toxicity profiling defined NOAEL and LOAEL values for 31 of the 37 test compounds (Supplementary Table 2). The remaining 6 compounds (lamotrigine, phenytoin, primidone, camphor, penicillin G, and metformin) did not exhibit developmental toxicity at any testable dose and therefore were only assigned a NOAEL value (Supplementary Table 2). The NOAEL value for these compounds is thus likely an underestimation of the true toxicity threshold. Most compounds (4/6) that could only be assigned a developmental NOAEL value also did not produce any adverse effects on *D. discoideum* growth.

We next used the fluorescence plate reader assay to validate the qualitative data (and vice versa). Where possible the highest developmentally toxic dose that did not exhibit growth toxicity was selected. For the small number of compounds that only exhibited developmental toxicity at doses greater than the growth toxicity NOAEL, the lowest developmentally toxic dose was used. For the 6 compounds that did not exhibit any developmental toxicity, the growth toxicity NOAEL dose was used. The data from the RFP reporter strains for the major stages of *D. discoideum* development (streaming, mound, slug, culminant, and fruiting body) was processed in several different ways (see Materials and Methods for details). First, we determined whether each marker exhibited a significant peak of expression, and if so when the peak level of expression occurred in comparison to control untreated samples. Together, these measurements allowed us to define whether there is block in development, or whether there is delay in the timing of development. In most cases, this quantitative data matched the qualitative data. Overall, this revealed identical observations in 128/167 (77%) cases. This increased to 135/167 (81%) when defects in timing and lack of expression were pooled (Supplementary Figure 4). Second, we also determined the level of expression of each marker at its peak in control and treated samples. This allowed us to also quantitatively determine if development was asynchronous, or partially blocked. When this additional phenotypic data were combined with the timing data 136/167 (82%) observations were in agreement between the qualitative and quantitative datasets (Supplementary Figure 4). Together, these observations reveal that qualitative and quantitative data are similar. Of the compounds not expected to cause developmental toxicity by qualitative observations (lamotrigine, phenytoin, primidone, acebutolol, camphor, penicillin G, and metformin), only one exhibited any defects in reporter gene activity. Similarly, all but one of the compounds expected to cause developmental toxicity (Vinclozolin) showed no effects with one or more developmental reporter (29/30). This suggests that the high-throughput assay can substitute for morphological observations and could allow *D. discoideum* to be used for DART testing in nonexpert laboratories.

#### Doses That Result in Toxicity in *D. discoideum* Correlate With Values in Other DART Models

If *D. discoideum* is to represent a model for developmental toxicity testing, then toxicity in this system should reflect toxicity in other DART models, as well as the effects seen in humans. The first indication that this may hold is that there are gross differences in NOAEL values between teratogenic and nonteratogenic compounds in *D. discoideum* growth and development (Supplementary Figure 5). Furthermore, most test compounds (27/37) caused developmental toxicity at the growth NOAEL dose (or lower); suggesting that development may provide a more sensitive readout for toxicity testing. Moreover, the scale of this difference is likely an underestimation because many nonteratogenic controls do not result in defects at the highest testable concentration (5/10 during growth, 3/10 during development). Previously, such differences in growth LOAEL and development toxicity NOAEL values have been quantitatively assessed to evaluate the ability of model systems to differentiate between known human teratogenic and nonteratogenic compounds (Ball *et al.*, 2014; Brannen *et al.*, 2010). Using this approach (see Materials and Methods), we found that when all 37 compounds were analyzed, the performance of *D. discoideum* is largely comparable to other alternative models (Ball *et al.*, 2014; Gustafson *et al.*, 2012; Leconte and Mouche, 2013; Panzica-Kelly *et al.*, 2013 and see Discussion) (Table 1). When the test compounds that could not be assigned a growth LOAEL due to limitations in solubility, the remaining 24 compounds showed similar trends (Table 1).

**Figure 2.**
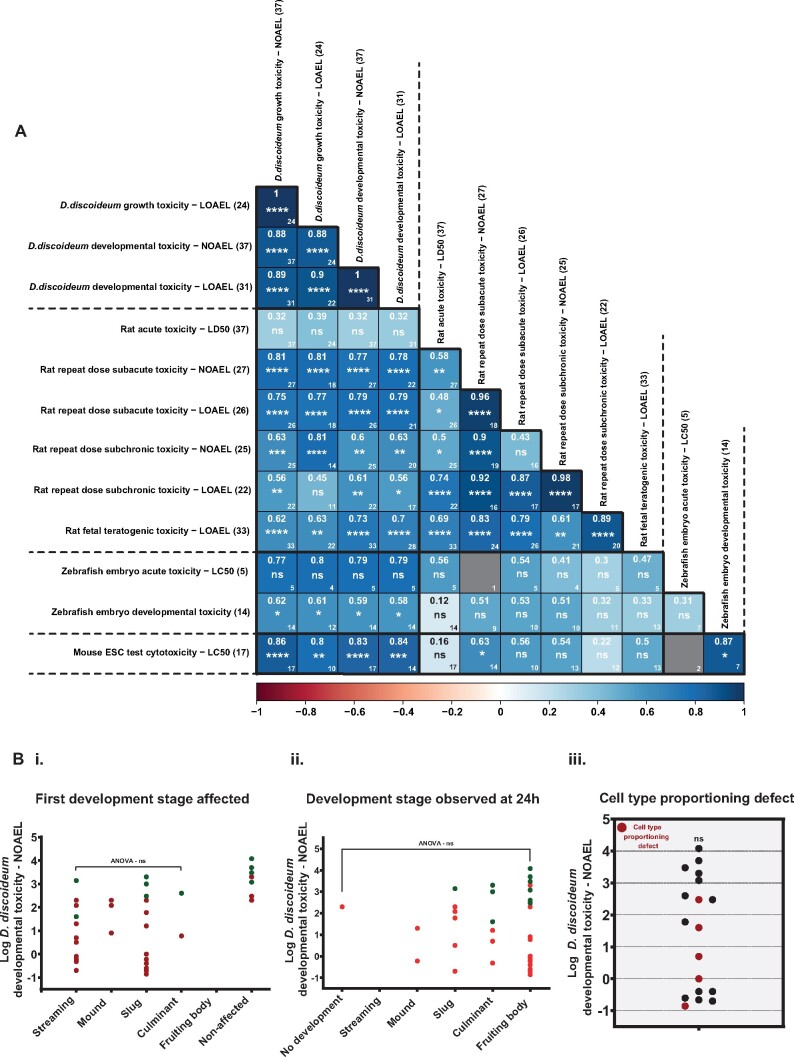
*Dictyostelium discoideum* toxicity is predictive of toxicity in rats and alternative testing models. A, A Pearson correlation matrix comparing toxicity endpoints from *D. discoideum*, rat, zebrafish embryo, and mouse embryonic stem cell models. Color scale shows strength of the correlation with darker blue indicating a stronger positive correlation. The *r* value is shown in the upper center of each box. Value in the lower right corner of each box shows the number of comparisons in each correlation. Statistically significant correlations were individually determined by Pearson correlation analysis. * indicates significance where *= <.05, **= <.01, ***=<.001, and ****=<.0001. Comparisons that contained too few values for significance testing are colored gray. The values after row and column headers indicate the number of the 37 test compounds where values could be obtained for each dataset. B, Developmental toxicity NOAEL does not predict developmental phenotypes. *D. discoideum* developmental toxicity NOAEL values (µM) do not predict developmental toxicity phenotypes (i) the first developmental stage affected (ANOVA—*p* = .58), (ii) the developmental stage observed after 24 h (ANOVA—*p* = .71), and (iii) cell type proportioning defect (*t* test—*p* = 0.204).

To further define the relationship between the effects seen in *D. discoideum* to those in higher organisms, the dose that results in toxicity in different systems was compared for the 37 compounds. First, a literature search was conducted to collect rat toxicity data for all 37 compounds (see Materials and Methods). Where possible, NOAEL and LOAEL values were recorded for each compound in each dataset (Supplementary Table 3). Four *in vivo* rat toxicity value datasets were collected: acute toxicity (LD_50_ values), repeat dose (subacute and subchronic), and developmental toxicity. The *D. discoideum* growth and development toxicity NOAEL and LOAEL endpoints were found to significantly correlate with all but one (repeat dose subchronic—LOAEL) of the rat repeat dose datasets and fetal teratogenicity datasets (19/20) (Figure 2A). Although rat acute toxicity data correlate positively with the *D. discoideum* data, this relationship is not significant (Figure 2A). This discrepancy is likely due to the nature of acute toxicity studies, which are often skewed toward testing a small number of high doses (Erhirhie *et al.*, 2018). Finally, we also compared the *D. discoideum* data to other alternative model systems for which freely available data were available for a sufficient number of compounds (Supplementary Table 4). Despite, the smaller datasets, the *D. discoideum* growth and development toxicity values were again found to positively (and significantly) correlate to toxicity values from zebrafish embryo and mouse embryonic stem cell test assays (Figure 2A).

#### Growth and Developmental Toxicity Are Related in *D. discoideum*, but Development Provides a Richer Phenotype

Whilst there are clear differences between the doses that generate growth and developmental phenotypes for teratogenic and nonteratogenic compounds, these data also reveal a correlation between doses that affect growth and development (Figure 2A). It is logical to assume that this reflects the fact that components of molecular and metabolic pathways are used repeatedly through the life cycle of an organism, albeit at different times and in different tissues. Indeed, a similar relationship can also be seen between different datasets collected from other organisms (Figure 2A). However, toxicity during development is not simply a proxy for growth. For example, when the first stage of development affected in *D. discoideum* is compared with the development NOAEL, there is no significant relationship between dose and phenotype (Figure 2Bi). This breakdown in the relationship between dose and phenotype is also seen when the terminal developmental stage at 24 h is used as a phenotypic measure (Figure 2Bii). Finally, we also assessed whether more subtle developmental defects such as changes in prestalk and prespore marker expression at the fruiting body stage could provide additional phenotypic discrimination. The expression of the prespore and prestalk reporter markers was compared with wild-type at 24 h. In order to account for artifacts that might arise due to developmental delay (eg, prespore expression increases at an earlier stage than wild-type), only those compounds for which fruiting body marker expression was normal were included. For several of the tested compounds, significant changes in prestalk/prespore proportioning could be seen (Figure 2Biii). Again, no relationship between dose and phenotype was evident, thus indicating that defects in cell type proportioning can help provide a richer phenotype that may be used to help understand developmental toxicity.

#### *D. discoideum* Can Be Used to Provide a Genetic Phenotype

**Figure 3.**
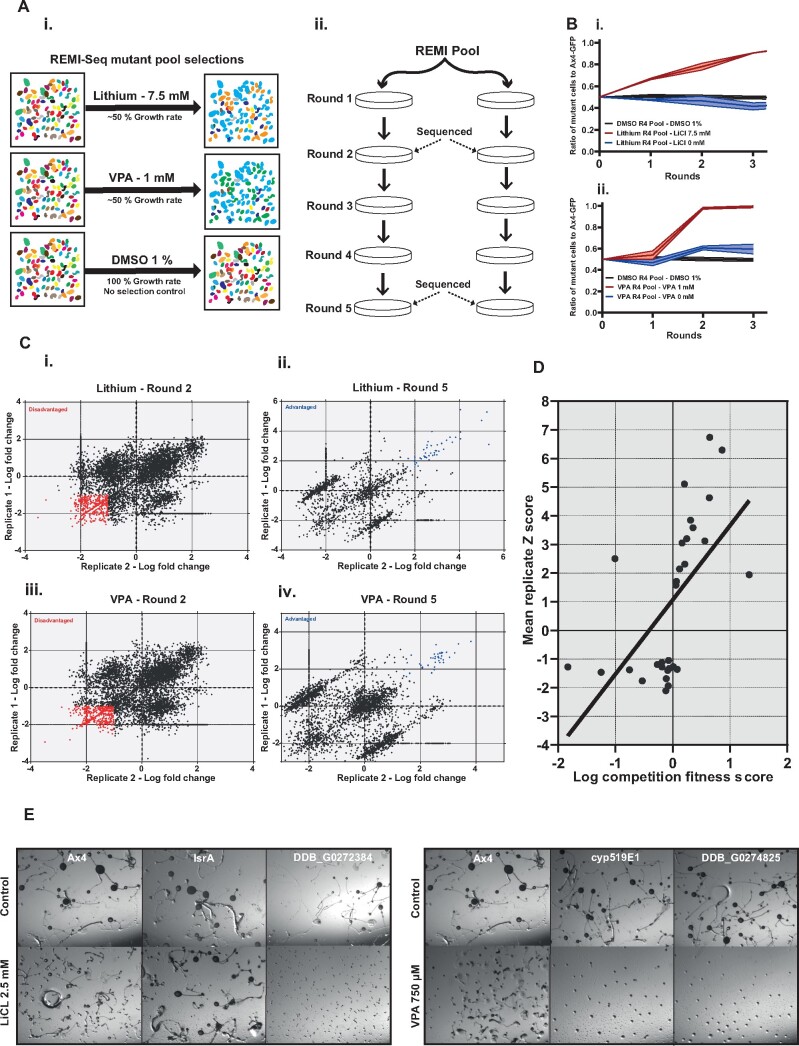
A parallel phenotype approach to compare the toxic effects of lithium and VPA. A, Schematic of the REMI-Seq screening method. (i) REMI-Seq mutants were treated with either 7.5 mM LiCl, 1 mM VPA, or 1% DMSO. (ii) The LiCl, VPA, and DMSO screens were conducted in duplicate, with cells grown in log growth phase over 5 rounds. Samples were taken for sequencing at rounds 2 and 5. B, Round 5 mutant pools are enriched for resistant mutants. REMI-mutant cells from round 4 of the lithium and VPA screens increase in frequency in the presence of each compound (red). In the absence of LiCl or VPA, the pools (blue) behave similarity to the DMSO control cells (black) (mean ± SD, *n* = 2). C, Selection of significantly advantaged and disadvantaged lithium and VPA mutants. Correlation of the log fold change of the read counts for each mutant compared with mean DMSO control values in the lithium (i, ii) and VPA (iii, iv) screens. *Z* score was used to identify significant outliers. Disadvantaged mutants are shown as red and advantaged mutants are shown as blue. D, Independent validation of advantaged and disadvantaged mutants. Competition assays were performed on 30 individual REMI mutants selected from the lithium and VPA advantaged and disadvantaged mutant lists. A competition fitness score was calculated from the relative performance of each mutant in the presence or absence of the relevant screen drug. This was compared with the mean *Z* score from the REMI-Seq experiment using Pearson correlation analysis (*r* = 0.58, *p* =.0007). E, A subset of validated mutants exhibit altered responsiveness to lithium and VPA in development. Ax4 and a selection of validated lithium and/or VPA advantaged and disadvantaged mutant cells were developed for 72 h in the presence of a range of lithium and VPA doses. Images are representative of multiple independent experiments and show examples of mutants that display resistance or sensitivity.

A key goal of developmental toxicity testing is to integrate phenotypic information with information about the cellular, molecular, and genetic effects of different compounds (Guengerich and MacDonald, 2007). This would allow for better comparisons between different molecules and a greater understanding of why they exert therapeutic and/or toxic developmental effects. We therefore tested whether this could be achieved by using REMI-Seq technology (Gruenheit *et al.*, 2021) to screen a library of genetic variants of *D. discoideum* to identify mutants that exhibit resistance or hypersensitivity to chemical toxicity. Given the simplicity of measuring toxicity during growth, as well as the correlation between effects on cell growth and development (Figure 2A), growth offers advantages for such an assay. Furthermore, although this relationship breaks down when more specific developmental phenotypes are measured (Figure 2B), we reasoned that it is logical to assume that this would also be true if more specific processes which are required for growth were assayed instead (eg, nutrient uptake vs cell division machinery). Therefore, to assess the utility of REMI-Seq as a “genetic phenotyping” tool for toxicity evaluation and categorization in *D. discoideum* proof of principle screens were performed with lithium and VPA, which are both therapeutic mood stabilizers and mammalian teratogens (Yu and Greenberg, 2016). Furthermore, they are known to share biological targets (eg, phosphoinoistol signaling), although the full extent of this overlap is unknown (Yu and Greenberg, 2016). Lithium and VPA also cause developmental toxicity in *D. discoideum* (Tillner *et al.*, 1998; Williams *et al.*, 1999). Because toxicity during growth or development are related (Figure 2A), we reasoned that vegetative growth could be used in REMI-Seq screens to identify mutants with altered responses to lithium or VPA. Indeed, we found that 10 mM lithium affects the growth of known developmentally resistant mutant (*dpoA^-^*) to a significantly lesser extent than wild-type cells (data not shown) (Williams *et al.*, 1999). Next, we identified concentrations of lithium and VPA that would provide a moderate selective pressure for each compound in order to ensure that the full spectrum of resistant or sensitive mutants could be identified. The relative growth rate of Ax4 cells was tested over 48 h in different concentrations of lithium and VPA. From this, concentrations of 7.5 mM for lithium and 1 mM for VPA were selected for REMI-Seq screening (Figure 3Ai).

A REMI-Seq mutant pool containing approximately 21 000 mutants was grown up in the presence of either 1 mM VPA or 7.5 mM lithium (Figure 3Ai). As a control, the pool was also grown in 1% DMSO in order to identify and eliminate mutants that simply grow more quickly or slowly (Figure 3Ai). Each screen was conducted in duplicate and continued until the time taken for plates to reach confluence in the presence of lithium and VPA had decreased (5 rounds) (Figure 3Aii). To ensure that this was because resistant (advantaged) mutants had increased in frequency, the growth rate of each pool was measured. Competition assays were performed between a GFP expressing fluorescent wild-type strain and pooled cells from round 4 of the lithium, VPA, or DMSO screens. The relative proportion of labeled to unlabeled cells was scored by flow cytometry. Both the lithium and VPA round 4 pools increased in proportion to wild-type cells when exposed to the screen doses of each compound (Figure 3Bi, ii). This did not occur in the absence of lithium or VPA treatment, which suggests that we have not simply selected for faster growing mutants. This idea is supported by the fact that the control DMSO screen did not change in relative frequency (Figure 3B). These results suggest that the later rounds of the lithium and VPA screens are enriched for resistant mutants. However, if enriched “advantaged” mutants rise in frequency, this necessitates that other mutants will decrease in frequency and drop out. Consequently, it becomes difficult to distinguish neutral mutants from hypersensitive or “disadvantaged” mutants, which had dropped out of the pool more quickly than expected. This problem is less severe at earlier rounds when advantaged mutants have not had sufficient time to take over the pool. Consequently, in addition to sequencing gDNA from round 5 to identify advantaged mutants, gDNA was sequenced from round 2 in order to preferentially identify disadvantaged mutants (Figure 3Aii).

gDNA samples from rounds 2 and 5 were sequenced, which yielded approximately 450- and 490 million reads respectively (Supplementary Table 5). Approximately 60% of the reads could be mapped to a genomic REMI insert loci as described by Gruenheit *et al.* (2021) (Supplementary Table 5). After mapping the sequenced reads, the normalized read counts of each biological replicate were compared with assess the quality and reproducibility of the data (Supplementary Figure 6A). Biological replicates were highly correlated (*p* = <.0001) (Supplementary Figure 6A). As expected, round 5 of the lithium and VPA screens contained fewer mutants than at round 2, or either round of the DMSO control (Supplementary Figure 6A). We next identified significantly advantaged (round 5) and disadvantaged (round 2) mutants in the lithium and VPA screens by comparing the relative abundance of every mutant to its abundance in the DMSO screen (Figure 3C). Because the DMSO screen has gone through the same number of generations as each selection, it allows growth mutants to be removed. Mutants were first divided into 3 bins depending on their mean normalized readouts in the DMSO screen (Supplementary Figs. 6B and 6C). This allows mutants with similar read counts to be compared. Most importantly, it ensures that mutants with <100 read counts, which have a high stochastic dropout rate, are treated separately (Gruenheit *et al.*, 2021). The relative fold change of each mutant in each bin was calculated for each replicate of the lithium and VPA screens. Next, we identified significantly advantaged and disadvantaged mutants. Mutants with a *Z*-score of >1.5 in both replicates of round 5 were defined as advantaged (Figure 3Cii, iv). A less stringent threshold of −1 was used to identify disadvantaged mutants as only mutants from the bins 1000 and 10 000 were considered (Figure 3Ci, iii). After defining advantaged and disadvantaged mutants, intragenic insertions mutations were assigned to genes. In addition, promoter insertions were assigned to their corresponding gene. Finally, to ensure that all inherent growth mutants had been removed, we compared our mutant lists to previously reported axenic growth mutants (Gruenheit *et al.*, 2021). No previously characterized axenic advantaged mutants were found in our lists, but a small number of axenic growth disadvantaged mutants were removed.

In order to experimentally validate our method of identifying mutants with advantages or disadvantages to lithium or VPA exposure, the growth rates of a subset of mutants was measured in competition with wild-type cells. Individual mutants were obtained from an independently generated REMI-Seq collection that contains many of the same mutations as the pooled library (or alternative alleles of the same mutants) (Gruenheit *et al.*, 2021). In total, we selected 30 mutants for validation from the round 2 and 5 mutant lists. We found that the majority of the mutants exhibited the expected behavior and recapitulated the advantaged or disadvantaged phenotype (Figure 3D). Finally, we tested whether mutants identified due to their effects on the susceptibility of cells to these compounds during growth also exhibit altered responsiveness to LiCl or VPA during the developmental cycle. In total 13 mutants from the validated round 2 and 5 mutant lists were assessed with a range of LiCl and/or VPA doses. We found that the effects of these compounds on development was altered (resistant or more sensitive) in the majority of these mutants (70%–14/20) (Supplementary Table 6) (Figure 3E). This is in agreement with the fact that there is a strong correlation between doses required to affect growth and development. Therefore, REMI-Seq which is most easily carried out during growth, can also be used to define and compare the gene networks affected by lithium and VPA during development.

#### Genes That Affect Responsiveness to Lithium or VPA Toxicity Share Extensive Similarity

**Figure 4.**
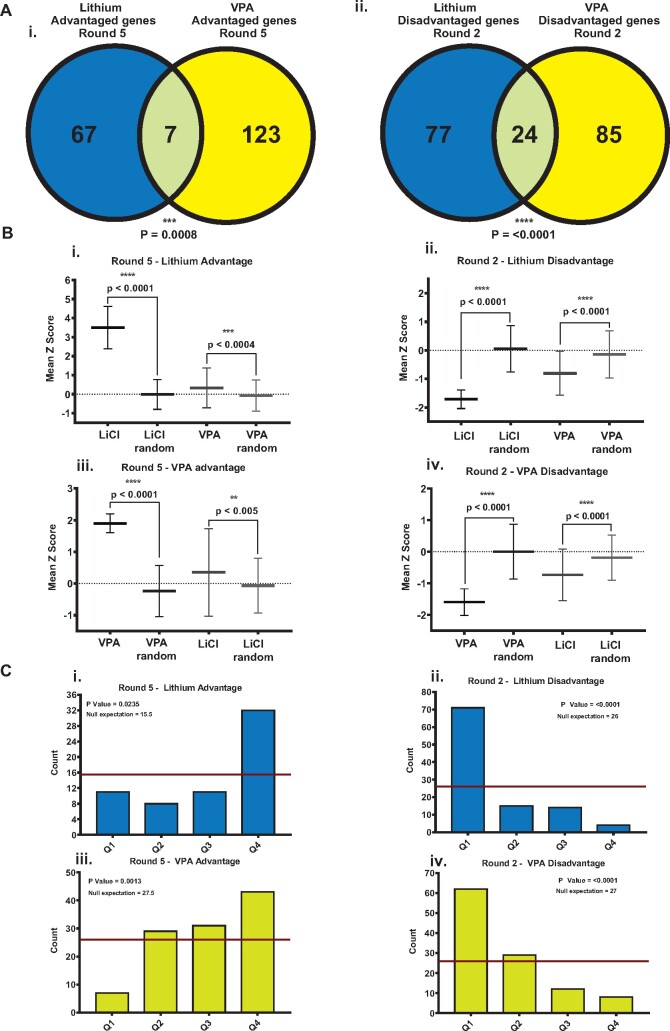
Many genes identified in the lithium and VPA screens are common. A, Advantaged and disadvantaged lithium and VPA genes significantly overlap. Hypergeometric distribution testing reveals a significant overlap in the genes identified from each screen (*p* = .0008, <.0001). B, The behavior of LiCl and VPA advantaged and disadvantaged mutants is similar in each drug. The behavior (*Z* score) of mutants identified in each screen was compared with a random sample of the same number of mutants. Mutants were randomly sampled 10× (mean ± SD). C, Rank testing reveals there is strong bias for mutants to have similar phenotypes in both screens. Mutants in the lithium and VPA screens were ranked according to their mean *Z* score. The ranked mutants were split into quartiles with Q1 containing the most decreased mutants and Q4 the most increased. Mutants from the lithium and VPA advantage (i, iii) and disadvantage lists (ii, iv) were assessed as to where they ranked in the other screen. Significant enrichment in any of the quartiles was assessed using a Chi-squared test.

A number of genes and pathways that are affected by lithium and/or VPA have been described. However, the degree to which these targets are specific or shared is unclear (Yu and Greenberg, 2016). We thus tested whether REMI-Seq can be used to reveal these similarities and differences, and therefore provide an unbiased genetic phenotype. We found that there is a statistically significant overlap between lithium and VPA growth advantaged or growth disadvantaged genes (Figure 4A). In fact, this is likely an underestimation of the overlap, as the gene lists were created using stringent cutoffs and a high reproducibility. This is evident if the mean *Z* scores of the significantly advantaged or disadvantaged mutants in lithium or VPA are compared. Disadvantaged mutants were generally found to have a lower-than-average Z-score in both selections (Figure 4Bii, iv). A similar trend was observed for advantaged mutants, as their average *Z* score was significantly higher than expected (Figure 4Bi, iii). Consequently, when rank ordered according to *Z* score, disadvantaged and advantaged mutants are significantly enriched in the lower or upper quartiles of the other screen, respectively (Figure 4C). To directly test this, the growth of a number of VPA and lithium-advantaged mutants were compared in the other compound. These results reveal that many of the mutants exhibit cross resistance, although the magnitude of the resistance was lower in the other compound (Supplementary Figure 7). These results suggest that REMI-Seq can be used to compare the mechanism of action of different compounds. Furthermore, they suggest there is a strong mechanistic link between the toxic effects of lithium and VPA.

#### Gene Ontology Analysis Reveals Biological Processes Affected by Lithium and VPA Toxicity

**Figure 5.**
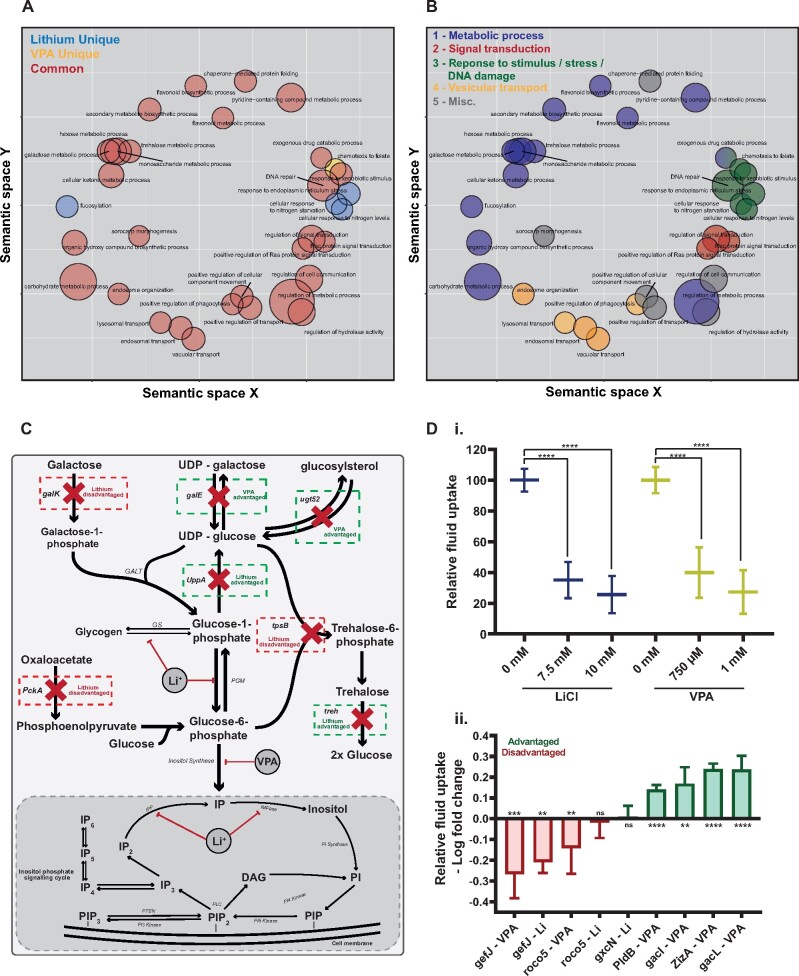
GO term analysis reveals that lithium and VPA mediate cellular toxicity via common biological processes. A, Significantly overenriched biological process GO terms are shared between lithium and VPA. The refined GO terms from the lithium, VPA, and “combined” GO term analysis were plotted on semantic space using the REVIGO tool (Supek *et al.*, 2011). GO terms with biological similarities are plotted closer together. The size of each circle represents the biological complexity of the term, with larger circles representing “broader” terms. Each term is classified as either lithium unique, VPA unique or common between the two, dependent on whether the genes underlying the GO terms came from the lithium or VPA screen. Plots are colored: blue for uniquely lithium, gold for uniquely VPA, or red for common between the two. B, GO term analysis reveals key biological processes affected by lithium and VPA toxicity. The overenriched biological process GO terms can be classified into 4 key processes: metabolic process (blue); signal transduction (red); response to stimulus/stress/DNA damage (green), and vesicular transport (yellow). Miscellaneous GO terms are colored gray. C, Lithium and VPA metabolic process genes are closely linked to inositol biosynthesis and the inositol phosphate signaling cycle. A schematic of the metabolic pathways and metabolic genes from the 3 most significantly overrepresented metabolic process GO terms: “galactose metabolic process,” “monosaccharide metabolic process,” and “trehalose metabolic process.” D, Lithium and VPA treatment significantly reduces cellular fluid uptake. (i) At both a 7.5- and 10-mM dose of LiCl and a 750-μM and 1-mM dose of VPA a significant reduction in cellular fluid uptake was observed in Ax4 cell line (*t* test, mean ± SD). (ii) When treated with either lithium (7.5 mM) or VPA (1 mM), most mutants assayed from the signal transduction classification of GO terms uptake significantly more or less fluid in comparison to a control mutant dependent on whether they are advantaged or disadvantaged respectively (*t* test, mean ± SD).

The overlap between the genes identified by REMI-Seq suggests that the mechanism(s) by which lithium and VPA exert their effects are similar. To further test this idea, and to better understand their mode of action, GO term analysis was performed using the GSEAbase R package (Morgan *et al.*, 2021) and the REVIGO tool (to remove redundant GO terms) (Supek *et al.*, 2011). To assign significance to overenrichment, gene lists were compared with a gene universe of 6161 genes based on all detectable mutants in any of the sequenced REMI-Seq libraries (Gruenheit *et al.*, 2021). Thirty-four enriched GO terms were plotted onto arbitrary semantic XY axes (Supek *et al.*, 2011) (Figure 5A). The majority of GO terms contained genes identified in either screen (Figure 5A). These data support the idea that there is a close mechanistic link between the effects of lithium and VPA. Furthermore, when plotted in this way, 4 clusters of GO terms can be seen. Manual curation revealed these represent 4 discreet branches of the Biological Processes GO term tree (Supek *et al.*, 2011); “metabolic process,” “signal transduction,” “response to stimulus/stress/DNA damage,” and “vesicular transport” (Figure 5B).

Inositol depletion and the subsequent attenuation of inositol phosphate signaling has previously been shown to explain some of the physiological effects of lithium and VPA (Yu and Greenberg, 2016). In support of this idea the most significantly enriched GO term within the metabolic process group was “galactose metabolism” (Figure 5B). This contains 3 out of 4 enzymes in the Leloir pathway (*galK*, *uppA*, and *galE*), which is ubiquitous among eukaryotes and functions to covert galactose into metabolically active glucose-6-phosphate (Holden *et al.*, 2003). Glucose-6-phosphate (and glucose-1-phosphate) are metabolic precursors for the *de novo* synthesis of inositol (Figure 5C). *galK*, *uppA*, and *galE* all play a role in the production or breakdown of glucose-1-phosphate (Figure 5C). Similarly, other genes within the metabolic process group such as tpsB and treh (“trehalose metabolism”) and pckA and ugt52 (“monosaccharide metabolic process”) were also found to be metabolically adjacent to glucose-1-phosphate or glucose-6-phosphate (Figure 5C).

One consequence of inositol depletion is a fall in PIP_3_ levels (Teo *et al.*, 2009). PIP_3_ is key signaling molecule required for macropinocytosis in *D. discoideum* (Veltman *et al.*, 2014). Because macropinocytosis is the primary mechanism by which *D. discoideum* cells take up nutrients in liquid growth medium, this could represent a common mechanism by which both VPA and lithium exert their growth toxicity effects (Xu *et al.*, 2007). Indeed, we find that the concentrations of lithium and VPA used in the screens both significantly reduce fluid uptake (Figure 5Di). Macropinocytosis also requires Ras and Rac proteins to be recruited to membrane-bound signaling patches of PIP_3_, which in turn recruit downstream activators to trigger actin polymerization and fluid uptake (Williams *et al.*, 2019). Interestingly, the “signal transduction” group of GO terms includes both “rac” and “ras” protein signal transduction GO terms (Figure 5B). We therefore assessed whether changes in fluid uptake dynamics may underlie the behavior of these mutants. When representative signal transduction mutant cells were compared with wild-type cells, most were found to affect fluid uptake (Figure 5Dii). These results suggest that lithium and VPA toxicity impacts macropinocytosis, likely due to changes in phosphoinoistol and ras/rac signaling. These results, together with the analysis of the metabolism process GO terms, demonstrate the proof of principle that REMI-Seq could provide a tool to understand the molecular genetic pathways underpinning drug action and toxicity.

## DISCUSSION

There are many different desirable features for alternative developmental toxicity models. These include the ability to predict mammalian *in vivo* toxicity, amenability to high-throughput approaches, low cost of maintenance, ability to understand the molecular genetic effects of different compounds and sufficient biological complexity to reflect the teratogenic effects seen in humans (Brannen *et al.*, 2016). It is likely that no single system that can meet all these criteria and that a battery of systems will need to be used in any pipeline. Our results suggest that *D. discoideum* may prove a useful system to complement those previously described. The relative success with which *D. discoideum* is able to classify compounds as teratogenic (or nonteratogenic), as well as reflecting the dependence of developmental toxicity on exposure levels provides one such measure (Daston *et al.*, 2014). *Dictyostelium discoideum* has an overall concordance with mammalian *in vivo* classification of 69% across all 37 test compounds (Table 1). This is comparable to figures reported for other established alternative DART testing models. Initial work on Zebrafish using a smaller selection of test compounds reported 85% overall concordance (Gustafson *et al.*, 2012), which was followed up with 65% and 82% reported overall concordance when a larger cohort of test compounds were tested across 2 independent laboratories (Ball *et al.*, 2014). A modified mouse embryonic stem cell assay reported a 72% predictivity against a test cohort of 65 compounds (Panzica-Kelly *et al.*, 2013). Consequently, *D. discoideum* correctly identifies nonteratogens in 80% of cases (ie, false positive rate of 20%) across all 10 test nonteratogenic compounds [Table 1]) which is comparable to the widely used zebrafish system (16%–32%) (Ball *et al.*, 2014). Furthermore, despite requiring concentrations of >200µM on average to induce developmental toxicity (Supplementary Table 1), this only results in false negative teratogen reporting rate of 33%, which is comparable to other systems (eg, 42%–66% in zebrafish). Our initial results are therefore encouraging, but more test compounds will need to be assessed in the future including a greater number of nonteratogenic control compounds will be required to truly determine the overall accuracy of this system for predicting teratogenicity, and to identify types of compounds where it may fail. Indeed, such differences are likely given the fact that there may be species specific differences in toxic compound metabolism between *D. discoideum* and higher order mammalian species including humans (Zhang and Tang, 2018).

The *D. discoideum* growth and developmental toxicity assays developed for this study were designed to allow high throughput, simple, and quantitative evaluation of test compounds, with the potential to be further scaled up in an industrial setting. The microscopy-based assay provides a simple readout for effects on growth, yet reflects the interplay of numerous complex biological processes. However, alternative toxicity endpoints such as changes in cell morphology or cell motility, which have previously been used to assess chemical-mediated changes in *D. discoideum*, could also easily be conducted (Cocorocchio *et al.*, 2016; Liao and Kimmel, 2017). We have also shown that manual observation of developmental progression provides a simple method to assess developmental toxicity. However, manual observations are subjective, low throughput, and only amenable to trained researchers. Consequently, we also developed an automated development assay based on a set of fluorescent reporter strains that provide information about developmental progression. Analyses of these data does not require expertise in *D. discoideum* developmental morphology. This is important because one of the problematic aspects of current zebrafish developmental toxicity research has been to unify and harmonize the guidelines for testing procedures and scoring systems (Beekhuijzen *et al.*, 2015; Cassar *et al.*, 2019). Furthermore, scoring systems can still require expert analysis (Brannen *et al.*, 2010; Panzica-Kelly *et al.*, 2010; Weigt *et al.*, 2011). The fluorescence plate reader assay reported here avoids this as it generates quantitative statistical data which corresponded to “expert” observations of developmental toxicity.

In mammalian systems, examining morphological phenotypes caused by exposure to a developmentally toxic compound is the most common method to assess toxicity (Parasuraman, 2011). However, many toxic effects result in subtle phenotypes (which are difficult to detect) or phenotypic outcomes that are unobservable (such as adult behavioral changes) (Parasuraman, 2011). Furthermore, by only assessing the final morphological outcomes, the molecular initiating events and subsequent events that mediate developmental toxicity are hidden. Thus, simple phenotypic scoring of (possibly) complex biological processes inevitably results in reductive measurements of developmental toxicity. However, as developmental toxicity testing accounts for a large proportion of compliance failure, there is a pressing need to understand the targets and molecular mechanisms affected by compounds that fail (or succeed) in developmental toxicity testing (Guengerich and MacDonald, 2007). The field of toxicogenomics, attempts to link toxicity (including developmental toxicity) with genetic data (Alexander-Dann *et al.*, 2018). By comparing toxicity-mediating genes associated with novel compounds to characterized teratogenic compounds, common genetic markers of teratogenic phenotypes could be identified. Furthermore, as researchers develop new bioactive compounds they could quickly establish whether: all compounds in the same class cause developmentally adverse effects; specific chemical structure(s) result in developmental toxicity; and whether the teratogenic effects are a result of off-target events (Brannen *et al.*, 2016). To date, transcriptomics has been the most widely used method within this field, allowing changes in transcription profiles to be measured after toxic chemical exposure, which in turn can be used to identify gene networks associated with toxic mechanisms of action. Such studies have been invaluable for toxicity characterization and comparison, as they provide a transcriptional (rather than morphological) phenotype (Shankar *et al.*, 2019; Zheng *et al.*, 2018). Our results suggest that the *D. discoideum* REMI-Seq method could provide an additional toxicogenomic tool (Gruenheit *et al.*, 2021). REMI-Seq generates a genetic “phenotype” to evaluate developmental toxicity. This phenotype is detailed, but unbiased, and could potentially be used to classify developmentally toxic compounds. We were able to use REMI-Seq to evaluate the relationship between the effects of lithium and VPA. Despite some knowledge of the molecular mechanism of action of both compounds, the extent to which they are mechanistically related was uncertain (Yu and Greenberg, 2016). The REMI-Seq parallel phenotyping method was able to identify loci associated with the toxic effects of both compounds. This revealed a significant overlap between the lithium and VPA gene lists, as well as identifying targets and modes of action. This enabled us to develop novel hypotheses which could be experimentally tested. It is important to recognize that only 2 compounds with a known relationship were screened in this initial work. Consequently, it will be important to establish the effectiveness of the REMI-Seq method on a greater number of compounds, as well as a range of compound classes. Such work will add to the evidence presented in this study that *D. discoideum* represents a promising system to add to the battery of alternative developmental toxicity evaluation assays.

## SUPPLEMENTARY DATA

Supplementary data are available at *Toxicological Sciences* online.

## FUNDING

Wellcome Trust Investigator Award (095643/A/11/Z to C.R.L.T); a Wellcome Trust Biomedical Resource (101582/Z/13/Z to C.R.L.T.); a Wellcome Trust Institutional Support (105610/Z/14/Z to C.R.L.T.); a BBSRC CASE PhD to R.B. in partnership with Syngenta.

## AUTHOR CONTRIBUTIONS

R.P.B. performed all experiments, data analyses, and wrote the manuscript. K.W. and C.R.LT. conceived the project, helped analyze data, and wrote the manuscript. All correspondence and requests for materials should be addressed to C.R.L.T.

## DECLARATION OF CONFLICTING INTERESTS

The authors declared no potential conflicts of interest with respect to the research, authorship, and/or publication of this article.

## Supplementary Material

kfab097_Supplementary_Data
