# Supplementary material for: Dictyostelium discoideum: An Alternative Nonanimal Model for Developmental Toxicity Testing
Source: Toxicol Sci. 2021 Aug 13;183(2):302–18. doi: 10.1093/toxsci/kfab097 (PMC8538044; doi:10.1093/toxsci/kfab097)
Supplement: kfab097_Supplementary_Data [file kfab097_supplementary_data.zip › toxsci-21-0117-File008.docx]

**Supplemental Material**

***Dictyostelium discoideum:* an alternative non-animal model for developmental toxicity testing**

Robert P. Baines*^1^*, Kathryn Wolton^2^, Christopher R. L. Thompson*^1*^*

*^1^ Centre for Life's Origins and Evolution, Department of Genetics, Evolution and Environment, University College London, Darwin Building, Gower Street, London, WC1E 6BT, UK*

*^2^ Syngenta, Jealott’s Hill International Research Centre, Bracknell, Berkshire, RG42 6EY*

**Supplementary Figures**

**Figure S1. Test compound selection and physical characteristics.**

**A. Schematic of the three phases of compound selection.**

**B. The structural and physical composition of the test teratogenic and non-teratogenic groups are similar.** The molecular weight **(i)**, calculated structural complexity **(ii)** and polar surface area **(iii)** are not significantly different between the teratogenic and non-teratogenic test compounds (P = 0.52, 0.85, 0.13 respectively). **(iv)** The teratogenic and non-teratogenic compounds partition coefficient (CLogP) is significantly different (P = 0.0137) although the range of values widely overlaps (Determined by unpaired T-test).

**Figure S2. Development and optimisation of a time-lapse microscopy-based growth assay for *D. discoideum*.**

**A. Schematic of the growth assay.** An automated microscope with programable X and Y stage permits the growth of *D. discoideum* cells to be monitored in the presence of multiple compounds and/or doses simultaneously.

**B. Cells can be automatically recognised and counted.**

DIC images are inverted to increase the contrast between cells and background in order to automatically detect and count cells.

**C. Well film positioning does not affect cell doubling times.** Cell population doubling rates associated with five separate locations **(i)** within a 24 well exhibit no significant difference **(ii)** (ANOVA – P = 0.78)**.**

**D. Reducing the framerate under increases error in growth measurement.** Sequentially removing frames from a triplicate set of films shot with 7.5-minute frames results in an increase in error with a frame rate greater than 1 hour.

**Figure S3. A quantitative high throughput method to measure developmental toxicity in *D. discoideum***

**A. Developmental expression profiles of stage and cell type reporter genes. (i)** The developmental expression profile of six genes with peaks of expression that coincide with the major stages of development. **(ii)** The developmental expression profile of a prestalk marker – ecmA (red) and a prespore marker – pspA (green). Expression data from (Rosengarten *et al.*, 2015).

**B. Quantification of fluorescence readout from stage and cell type specific reporter genes.** Six developmental stage reporter strains **(i, ii, iii, iiii, v, vi)** were developed within a fluorescence plate reader the timing and level of fluorescence reflects the mRNA expression profiles of each gene. The fluorescence profiles of the cell type reporter strains PspA-GFP **(viii)** and EcmAO-RFP **(vii)** was also quantified.

**Figure S4. Qualitative and quantitative *D. discoideum* developmental toxicity datasets are similar.**

**A. Timing of the major stages of development can be assessed with qualitative observation and a quantitative assay.** Heatmaps depicting effects of the test compounds on the timing of the streaming, mound, slug, culminate and fruiting body stages of development. Left map from qualitative observation and the right map from the fluorescence plate reader quantitative assay. Green – normal timing, yellow – delayed timing, red – stage not reached after 24 hours.

**B. Heterogeneous developmental progression can be assessed along with developmental timing.** Heatmaps depicting effects of the test compounds on both the timing of the streaming, mound, slug, culminate and fruiting body stages of development and the heterogeneity of development. Left map from qualitative observation and the right map from the fluorescence plate reader quantitative assay. Green – normal timing, light green – heterogenous developments, yellow – delayed timing, light red – delayed timing and heterogenous development, red – stage not reached after 24 hours.

**C. Qualitative and quantitative assessments of developmental toxicity are comparable.** Heatmaps depicting comparisons between the qualitative and quantitative measures of toxicity for developmental timing (left) and developmental timing and heterogeneity (right). Green – agreement between datasets, light green – defect seen in both data sets, red – non-agreement between datasets. Grey – comparison not possible.

**Figure S5. Non-teratogenic compounds are only toxic at a significantly higher dose than teratogenic compounds.**

The mean growth **(A)** and developmental toxicity **(B)** NOAELs for teratogenic and non-teratogenic test compounds are significantly different (P = 0.047 & 0.0014 respectively). Data represents the mean ± SD.

**Figure S6. Biological replicates of the lithium, VPA and DMSO REMI-Seq screens significantly correlate.**

**A. Correlations of the normalised readcounts for the two biological replicates of the lithium, VPA and DMSO screens.** The biological replicates for rounds two and five of the lithium, VPA and DMSO screens significantly correlate when tested using Pearson’s Correlation (All correlations - P= <0.0001).

**B. & C. Z - score analysis of binned lithium and VPA biological replicates.** The log foldchange of every mutants’ reads in the lithium and the VPA screen in comparison to mean DMSO readcount. The mutants from the lithium and VPA screens are binned in three bins 10,000, 1000 or 100. Across all bins significantly fewer mutants remain in the later round 5 in comparison to round 2 due to the advanced selection. The selected advantaged mutants are coloured blue and the disadvantaged mutants are coloured red.

**Figure S7.**

**Mutants originally classified as uniquely lithium or VPA advantaged are also weakly advantaged in the other compound.**

The REMI mutant cells (DDB_G0274981 – **Ai**, DDB_G0274825 – **Aii**, DDB_G0277245 – **Aiii**, gpt10 – **A iv**) increase in frequency in the presence of the compound from the screen they were identified from (Red). They also increase in frequency in the other compound to a lesser extent (orange). (Mean ± SD, n=2).

**Supplemental Tables**

**Table S1** Teratogenic and non-teratogenic test compounds.

**Table S2** *D. discoideum* growth and development endpoint toxicity values.

**Table S3** *In vivo* rat toxicity values.

**Table S4** Alternative DART model toxicity values

**Table S5** REMI-Seq samples read and tag numbers.

**Table S6** Developmental responses of selected mutants to lithium and VPA exposure

|  | **Compound Name** | **General Usage** | **Classification** | **Molecular Weight** | **Primary**  **Solvent** |
| --- | --- | --- | --- | --- | --- |
| 1 | **Methotrexate** hydrate | Anti-cancer | Teratogen | 454.44 | DMSO |
| 2 | **Pemetrexed** | Anti-cancer | Teratogen | 427.41 | H_2_O |
| 3 | **Lamotrigine** | Bi-polar, Epilepsy | Teratogen | 256.09 | DMSO |
| 4 | **Carbamazepine** | Bi-polar, Epilepsy | Teratogen | 236.27 | DMSO |
| 5 | **Phenytoin** sodium | Epilepsy | Teratogen | 274.25 | DMSO |
| 6 | **Primidone** | Epilepsy | Teratogen | 218.25 | DMSO |
| 7 | **Valproic Acid** sodium | Bi-polar, Epilepsy | Teratogen | 166.2 | H_2_O |
| 8 | **Lithium** chloride | Bi-polar disorder | Teratogen | 42.39 | H_2_O |
| 9 | **Acitretin** | Auto-Immune | Teratogen | 326.43 | DMSO |
| 10 | **13-*cis*-Retinoic Acid** | Acne | Teratogen | 300.44 | DMSO |
| 11 | **Retinoic Acid** | Acne | Teratogen | 300.44 | DMSO |
| 12 | **Bosentan** hydrate | Hypertension | Teratogen | 569.63 | DMSO |
| 13 | **Sitaxentan** sodium | Hypertension | Teratogen | 476.89 | H_2_O |
| 14 | **Bexarotene** | Anti-cancer | Teratogen | 348.48 | DMSO |
| 15 | **Cadmium Sulphate** monohydrate | Industrial Electrical Component | Teratogen | 208.47 | H_2_O |
| 16 | **Hydroxyurea** | Antineoplastic | Teratogen | 76.05 | H_2_O |
| 17 | **Cyclophosphamide** monohydrate | Anti-cancer | Teratogen | 279.1 | H_2_O |
| 18 | **Cisplatin** | Anti-cancer | Teratogen | 300.05 | H_2_O |
| 19 | **Lead (II) Acetate** trihydrate | Heavy Metal | Teratogen | 379.34 | Glycerol |
| 20 | **Clomifene** citrate salt | Estrogen Receptor Modulator | Teratogen | 598.08 | DMSO |
| 21 | **Raloxifene** hydrochloride | Estrogen Receptor Modulator | Teratogen | 510.04 | DMSO |
| 22 | **Finasteride** | Male Baldness Treatment | Teratogen | 372.54 | DMSO |
| 23 | **Vinclozolin** | Fungicide | Teratogen | 286.11 | DMSO |
| 24 | **Diethylstilbestrol (DES)** | Synthetic estrogen | Teratogen | 268.35 | DMSO |
| 25 | **Salicylic Acid** | Pain, Inflammation | Teratogen | 138.12 | H_2_O |
| 26 | **Nifedipine** | Anti-Angina, Anti-hypertensive | Teratogen | 346.33 | DMSO |
| 27 | **Warfarin** sodium | Anticoagulant | Teratogen | 330.31 | DMSO |
|  |  |  |  |  |  |
| 28 | **Metoclopramide** hydrochloride | Stomach medication | Non-teratogen | 336.26 | H_2_O |
| 29 | **Cefotaxime** sodium | Antibiotic | Non-teratogen | 477.45 | H_2_O |
| 30 | **Sulfasalazine** | Rheumatoid arthritis | Non-teratogen | 398.39 | DMSO |
| 31 | **Ascorbic acid** | Dietary vitamin | Non-teratogen | 176.12 | H_2_O |
| 32 | **Acebutolol** hydrochloride | Anti-Angina, Anti-hypertensive | Non-teratogen | 372.89 | H_2_O |
| 33 | **Camphor** | Multipurpose compound | Non-teratogen | 152.23 | Ethanol |
| 34 | **Citric acid** | Acidifier | Non-teratogen | 192.12 | H_2_O |
| 35 | **Penicillin G** sodium | Antibiotic | Non-teratogen | 356.37 | H_2_O |
| 36 | **Saccharin** sodium hydrate | Sweetener | Non-teratogen | 205.17 | H_2_O |
| 37 | **Metformin** hydrochloride | Diabetes treatment | Non-teratogen | 165.62 | H_2_O |

**Table S1. Teratogenic and non-teratogenic test compounds.**

|  | **Compound Name** | | **Max. Concentration Assayable** | **Growth toxicity - NOAEL** | **Growth toxicity - LOAEL** | **Developmental toxicity - NOAEL** | **Developmental toxicity - LOAEL** |
| --- | --- | --- | --- | --- | --- | --- | --- |
| 1 | | **Methotrexate** | 1 mM | 15 µM | 30 µM | 200 nM | 1 µM |
| 2 | | **Pemetrexed** | - | 20 µM | 40 µM | 480 nM | 2.4 µM |
| 3 | | **Lamotrigine** | 1 mM | 200 µM | 300 µM | 200 µM ***** | - |
| 4 | | **Carbamazepine** | 1 mM | 300 µM | 500 µM | 60 µM | 300 µM |
| 5 | | **Phenytoin** | 1 mM (Crystals >300 µM) | 300 µM ***** | - | 300 µM ***** | - |
| 6 | | **Primidone** | 2 mM | 2 mM ***** | - | 2 mM ***** | - |
| 7 | | **Valproic Acid** | - | 250 µM | 500 µM | 20 µM | 100 µM |
| 8 | | **Lithium chloride** | - | 5 mM | 7.5 mM | 200 µM | 1 mM |
| 9 | | **Acitretin** | 500 µM | 27.5 µM | 30 µM | 220 nM | 1.1 µM |
| 10 | | **13-*cis*-Retinoic Acid** | 1 mM (Crystals >17.5 µM) | 17.5 µM ***** | - | 140 nM | 700 nM |
| 11 | | **Retinoic Acid** | 1 mM (Crystals >10 µM) | 10 µM ***** | - | 400 nM | 2 µM |
| 12 | | **Bosentan** | 2 mM (Crystals >120 µM) | 120 µM ***** | - | 120 µM | 600 µM |
| 13 | | **Sitaxentan** | - | 400 µM | 500 µM | 3.2 µM | 16 µM |
| 14 | | **Bexarotene** | 500 µM (Crystals >5 µM) | 5 µM ***** | - | 1 µM | 5 µM |
| 15 | | **Cadmium Sulphate** | - | 15 µM | 25 µM | 600 nM | 3 µM |
| 16 | | **Hydroxyurea** | - | 225 µM | 250 µM | 200 µM | 1 mM |
| 17 | | **Cyclophosphamide** | 25 mM | 25 mM ***** | - | 200 µM | 1 mM |
| 18 | | **Cisplatin** | 30 µM | 30 µM ***** | - | 6 µM | 30 µM |
| 19 | | **Lead (II) Acetate** | - | 250 µM | 300 µM | 8 µM | 40 µM |
| 20 | | **Clomifene** | - | 250 nM | 500 nM | 250 nM | 1.25 µM |
| 21 | | **Raloxifene** | 500 µM | 20 µM | 25 µM | 800 nM | 4 µM |
| 22 | | **Finasteride** | 1 mM | 75 µM | 100 µM | 600 nM | 3 µM |
| 23 | | **Vinclozolin** | 2 mM | 10 µM | 15 µM | 400 nM | 2 µM |
| 24 | | **Diethylstilbestrol** | - | 2.5 µM | 5 µM | 200 nM | 1 µM |
| 25 | | **Salicylic Acid** | 14 mM | 2.5 mM | 3 mM | 120 µM | 600 µM |
| 26 | | **Nifedipine** | 1 mM | 50 µM | 75 µM | 5 µM | 25 µM |
| 27 | | **Warfarin** | 5 mM | 450 µM | 500 µM | 16 µM | 80 µM |
|  | |  |  |  |  |  |  |
| 28 | | **Metoclopramide** | - | 1.5 mM | 2.5 mM | 300 µM | 1.5 mM * |
| 29 | | **Cefotaxime** | 10 mM | 10 mM ***** | - | 2 mM | 10 mM ***** |
| 30 | | **Sulfasalazine** | - | 750 µM | 1 mM | 40 µM | 200 µM |
| 31 | | **Ascorbic acid** | 5 mM | 5 mM ***** | - | 1 mM | 5 mM * |
| 32 | | **Acebutolol** | 1.2 mM | 1.2 mM ***** | - | 1.2 mM | 6 mM |
| 33 | | **Camphor** | 5 mM | 3.5 mM | 4 mM | 3 mM ***** | - |
| 34 | | **Citric acid** | - | 3.5 mM | 4 mM | 400 µM | 2 mM |
| 35 | | **Penicillin G** | 12 mM | 12 mM ***** | - | 12 mM ***** | - |
| 36 | | **Saccharin** | 100 mM | 35 mM | 70 mM | 1.4 mM | 7 mM |
| 37 | | **Metformin** | 5 mM | 5 mM ***** | - | 5 mM ***** | - |
|  | | *** Highest dose assayable** | |  |  |  |  |

**Table S2. *D. discoideum* growth and development endpoint toxicity values**

| **Compound Name** | **Acute Toxicity - LD_50_** | **Repeat dose – Subacute -**  **NOAEL** | **Repeat dose – Subacute -**  **LOAEL** | **Repeat dose – Subchronic -**  **NOAEL** | **Repeat dose – Subchronic -**  **LOAEL** | **Rat Fetal teratogenicity – LOAEL** |
| --- | --- | --- | --- | --- | --- | --- |
| **Methotrexate** | 135 mg / kg | 0.06 mg / kg / d – 28 days | 5.6 mg / kg / d – 28 days | n/a | n/a | 0.2 mg / kg / d |
| **Pemetrexed** | 980 mg / kg | 0.285 mg / kg / d – 2 weeks | 0.85 mg / kg / d – 2 weeks | n/a | n/a | 1 mg / kg / d |
| **Lamotrigine** | 205 mg / kg | 11.5 mg / kg / d – 3 weeks | 46 mg / kg / d – 3 weeks | 10 mg / kg / d – 13 weeks | 30 mg / kg / d – 13 weeks | 10 mg / kg / d |
| **Carbamazepine** | 1957 mg / kg | > 100 mg / kg / d – 28 days | n/a | n/a | n/a | 200 mg / kg / d |
| **Phenytoin** | 1635 mg / kg | n/a | 50 mg / kg / d – 3 weeks | 300 ppm / d – 13 weeks | n/a | 100 mg / kg / d |
| **Primidone** | 1500 mg / kg | 500 mg / kg / d – 2 weeks | 900 mg / kg / d – 2 weeks | 20 mg / kg / d – 14 weeks | 40 mg / kg / d – 14 weeks | 120 mg / kg / d |
| **Valproic Acid** | 670 mg / kg | 250 mg / kg / d – 2 weeks | 500 mg / kg / d – 2 weeks | 378 mg / kg / d – 6 months | 400 mg / kg / d – 90 days | 100 mg / kg / d |
| **Lithium** | 1530 mg / kg | n/a | 1.46 mEq / L serum – 30 days | 12.8 mg / kg / d – 4 months | 25.6 mg / kg / d – 4 months | 100 mg / kg / d |
| **Acitretin** | 4000 mg / kg | 5 mg / kg / d – 4 weeks | 10 mg / kg / d – 4 weeks | 3 mg / kg / d – 13 weeks | n/a | 15 mg / kg / d |
| **13-*cis*-Retinoic Acid** | 4000 mg / kg | 15 mg / kg / d – 4 weeks | n/a | 40 mg / kg / d – 12 weeks | n/a | 30 mg / kg / d |
| **Retinoic Acid** | 2000 mg / kg | 5 mg / kg / d – 28 days | 15 mg / kg / d – 4 weeks | 4 mg / kg / d – 13 weeks | 14 mg / kg / d – 13 weeks | 2.5 mg / kg / d |
| **Bosentan** | 1000 mg / kg | 200 mg / kg / d – 28 days | 2000 mg / kg / d – 28 days | 15 mg / kg / d – 13 weeks | 45 mg / kg / d – 13 weeks | 75 mg / kg / d |
| **Sitaxentan** | 980 mg / kg | 80 mg / kg / d – 4 weeks | 120 mg / kg / d – 4 weeks | 20 mg / kg / d – 13 weeks | 50 mg / kg / d – 90 days | 20 mg / kg / d |
| **Bexarotene** | 1500 mg / kg | n/a | 10 mg / kg / d – 4 weeks | n/a | 5 mg / kg / d – 90 days | 4 mg / kg / d |
| **Cadmium Sulphate** | 280 mg / kg | 10 mg / kg / d – 28 days | 20 mg / kg / d – 28 days | 3 mg / kg / d – 3 months | 8.58 mg / kg / d – 12 weeks | 0.5 mg / kg / d |
| **Hydroxyurea** | 5000 mg / kg | 50 mg / kg / d – 2 weeks | 500 mg / kg / d – 2 weeks | n/a | n/a | 180 mg / kg / d |
| **Cyclophosphamide** | 100 mg / kg | n/a | 280 mg / kg / d – 4 weeks | 6 mg / kg / d – 12 weeks | 12 mg / kg / d – 12 weeks | 6.2 mg / kg / d |
| **Cisplatin** | 25.8 mg / kg | n/a | n/a | n/a | 1 mg / kg / d – 11 weeks | 0.25 mg / kg / d |
| **Lead (II) Acetate** | 450 mg / kg | n/a | 60 mg / kg / d – 4 weeks | n/a | 7.5 mg / kg / d – 14 weeks | 120 mg / kg / d |
| **Clomifene** | 5750 mg / kg | n/a | 0.5 mg / kg / d – 11 days | n/a | n/a | 8 mg / kg / d |
| **Raloxifene** | > 5000 mg / kg | 10 mg / kg / d – 4 weeks | n/a | 25 mg / kg / d – 6 months | n/a | 1 mg / kg / d |
| **Finasteride** | 418 mg / kg | n/a | 3 mg / kg / d – 2 weeks | 80 mg / kg / d – 12 weeks | n/a | 0.3 mg / kg / d |
| **Vinclozolin** | >10000 mg / kg | 66 mg / kg / d – 4 weeks | 180 mg / kg / d – 28 days | 4 mg / kg / d – 3 months | 22 mg / kg / d – 3 months | 25 mg / kg / d |
| **Diethylstilbestrol** | 3000 mg / kg | 20 ug / kg / d – 3 weeks | 40 ug / kg / d – 3 weeks | n/a | n/a | 10 mg / kg / d |
| **Salicylic Acid** | 891 mg / kg | n/a | 646.5 mg / kg / d – 28 days | 50 mg / kg / d – 17 weeks | 500 mg / kg / d – 17 weeks | 200 mg / kg / d |
| **Nifedipine** | 1022 mg / kg | 50 mg / kg / d – 4 weeks | n/a | 100 mg / kg / d – 13 weeks | n/a | 10 mg / kg / d |
| **Warfarin** | 59 mg / kg | 1 mg / kg / d – 21 days | 2 mg / kg / d – 7 days | n/a | 0.077 mg / kg / d – 13 weeks | 0.16 mg / kg / d |
|  |  |  |  |  |  |  |
| **Metoclopramide** | 750 mg / kg | n/a | n/a | 100 mg / kg / d – 13 weeks | n/a | n/a |
| **Cefotaxime** | 20000 mg / kg | 300 mg / kg / d – 30 days | 1000 mg / kg / d – 30 days | 400 mg / kg / d – 13 weeks | 800 mg / kg / d – 13 weeks | > 1200 mg / kg / d |
| **Sulfasalazine** | 15600 mg / kg | 600 mg / kg / d – 28 days | n/a | 200 mg / kg / d | 500 mg / kg /d | > 200 mg / kg / d |
| **Ascorbic acid** | 11900 mg / kg | 100,000 mg / kg / d – 14 days | n/a | 10,000 mg / kg / d – 10 weeks | 27.3 g/ kg / d – 10 weeks | n/a |
| **Acebutolol** | 980 mg / kg | > 500 mg / kg / d – 14 days | n/a | 75 mg / kg / d – 13 weeks | 225 mg / kg / d – 13 weeks | > 1000 mg / kg / d |
| **Camphor** | >10000 mg / kg | 1000 mg / kg / d – 7 days | 2000 mg / kg / d – 7 days | n/a | 464 mg / kg / d | > 1000 mg / kg / d |
| **Citric acid** | 3000 mg / kg | 4000 mg / kg / d – 2 weeks | 9300 mg / kg / d – 2 weeks | n/a | n/a | > 2500 mg / kg / d |
| **Penicillin G** | >2000 mg / kg | 2400 mg / kg / d – 2 weeks | n/a | 750 mg / kg / d – 13 weeks | 1500 mg / kg / d – 13 weeks | n/a |
| **Saccharin** | 14200 mg / kg | 30,000 mg / kg / d – 4 weeks | n/a | 20000 ppm / d – 13 weeks | n/a | n/a |
| **Metformin** | 1000 mg / kg | 200 mg / kg / d – 2 weeks | 1000 mg / kg / d – 2 weeks | 200 mg / kg / d – 13 weeks | 600 mg / kg / d – 13 weeks | > 600 mg / kg / d |

**Table S3. *In vivo* rat toxicity values.**

| **Compound Name** | **Zebrafish embryo Acute toxicity - LC_50_**  **(Ali et al, 2011)** | **Zebrafish embryo Developmental toxicity**  **(Ali et al, 2014 - LC_50_) + (Ducharme et al, 2015 - LOAEL)** | **Mouse EST cell viability – IC_50_ (Mean) (Panzica-Kelly et al, 2013)** |
| --- | --- | --- | --- |
| **Methotrexate** | **-** | **110 µM** | **173 nM** |
| **Pemetrexed** | **-** | **-** | **-** |
| **Lamotrigine** | **-** | **-** | **-** |
| **Carbamazepine** | **-** | **249 µM** | **-** |
| **Phenytoin** | **-** | **-** | **-** |
| **Primidone** | **-** | **-** | **-** |
| **Valproic Acid** | **-** | **140 µM** | **2.026 mM** |
| **Lithium** | **78.42 mM** | **6.992 mM** | **500 µM** |
| **Acitretin** | **-** | **-** | **-** |
| **13-*cis*-Retinoic Acid** | **-** | **-** | **90 nM** |
| **Retinoic Acid** | **-** | **0.213 nM** | **2.35 nM** |
| **Bosentan** | **-** | **-** | **-** |
| **Sitaxentan** | **-** | **-** | **-** |
| **Bexarotene** | **-** | **-** | **-** |
| **Cadmium Sulphate** | **60 µM** | **10.91 µM** | **-** |
| **Hydroxyurea** | **-** | **-** | **70.86 µM** |
| **Cyclophosphamide** | **6.37 mM** | **4.246 mM** | **1.762 mM** |
| **Cisplatin** | **-** | **-** | **-** |
| **Lead (II) Acetate** | **160 µM** | **79.44 µM** | **-** |
| **Clomifene** | **-** | **-** | **-** |
| **Raloxifene** | **-** | **-** | **-** |
| **Finasteride** | **-** | **-** | **-** |
| **Vinclozolin** | **-** | **29 µM** | **-** |
| **Diethylstilbestrol** | **-** | **-** | **-** |
| **Salicylic Acid** | **340 µM** | **197.9 µM** | **-** |
| **Nifedipine** | **-** | **-** | **-** |
| **Warfarin** | **-** | **-** | **833.46 µM** |
|  |  |  |  |
| **Metoclopramide** | **-** | **-** | **633.96 µM** |
| **Cefotaxime** | **-** | **-** | **> 1 mM** |
| **Sulfasalazine** | **-** | **-** | **935.82 µM** |
| **Ascorbic acid** | **-** | **-** | **230.59 µM** |
| **Acebutolol** | **-** | **-** | **659 µM** |
| **Camphor** | **-** | **1.18 mM** | **> 1.25 mM** |
| **Citric acid** | **-** | **-** | **-** |
| **Penicillin G** | **-** | **-** | **7903 µM** |
| **Saccharin** | **-** | **25.95 mM** | **> 10 mM** |
| **Metformin** | **-** | **-** | **> 1 mM** |

**Table S4. Alternative DART model toxicity values**

**Table S5. REMI-Seq samples read and tag numbers.**

| **Round 2** | | | | **Round 5** | | | |
| --- | --- | --- | --- | --- | --- | --- | --- |
| **Sample** | **Read number** | **Mapped tag number** | **Percentage tags** | **Sample** | **Read number** | **Mapped tag number** | **Percentage tags** |
| DMSO Rep.1 | 338,276,907 | 254,755,206 | 75 | DMSO Rep.1 | 91,757,730 | 39,788,687 | 43 |
| DMSO Rep.2 | 11,855,631 | 9,879,304 | 83 | DMSO Rep.2 | 176,310314 | 110,800,280 | 63 |
| Lithium Rep.1 | 16,889,054 | 12,496,226 | 74 | Lithium Rep.1 | 62,040,640 | 21,380,739 | 35 |
| Lithium Rep.2 | 33,121,738 | 24,865,993 | 75 | Lithium Rep.2 | 50,214,576 | 22,559,156 | 45 |
| VPA Rep.1 | 26,313,690 | 20,925,040 | 80 | VPA Rep.1 | 57,846,084 | 12,536,510 | 22 |
| VPA Rep.2 | 25,634,176 | 20,660,206 | 81 | VPA Rep.2 | 60,389,710 | 12,811,616 | 21 |
|  |  |  |  |  |  |  |  |
| **Total** | **452,091,196** | **343,581,975** | **-** | **Total** | **498,559,054** | **219,876,988** | **-** |

**Table S6. Developmental responses of selected mutants to lithium and VPA exposure**

| **Selected lithium mutants** | | | | **Selected VPA mutants** | | | |
| --- | --- | --- | --- | --- | --- | --- | --- |
|  | **Developmental Phenotype** | |  |  | **Developmental Phenotype** | |  |
| **Gene Name** | **Low dose (5 mM)** | **High dose (7.5 mM)** | | **Gene Name** | **Low dose (750 μM)** | **High dose (1 mM)** | |
| flsE | No difference | Resistant | | DDB_G0286363 | No difference | No difference | |
| gxcN | No difference | No difference | | gpt10 | No difference | Sensitive | |
| lsrA | Resistant | No difference | | DDB_G0274981_2_ | Sensitive | Sensitive | |
| DDB_G0286363 | Resistant | No difference | | DDB_G0274825 | Sensitive | Sensitive | |
| gpt10 | No difference | No difference | | Cyp519E1 | Sensitive | Sensitive | |
| DDB_G0284721_1_ | Resistant | Resistant | | lsrA | Sensitive | Sensitive | |
| DDB_G0274981_2_ | Sensitive | Sensitive | | nosip | Sensitive | No difference | |
| Cyp519E1 | No difference | No difference | | DDB_G0272923_1_ | No difference | Sensitive | |
| nosip | No difference | No difference | | DDB_G0293302 | No difference | No difference | |
| DDB_G0272923_1_ | No difference | No difference | |  |  |  | |
| DDB_G0272384_1_ | Sensitive | Sensitive | |  |  |  | |
| _1_Different (but neutral) phenotype in the absence of compound.  _2_Severve developmental phenotype in the absence of compound. | | | | | | | |
